# Pre-implantation exogenous progesterone and pregnancy in sheep. II. Effects on fetal-placental development and nutrient transporters in late pregnancy

**DOI:** 10.1186/s40104-021-00567-1

**Published:** 2021-04-08

**Authors:** Katherine M. Halloran, Emily C. Hoskins, Claire Stenhouse, Robyn M. Moses, Kathrin A. Dunlap, M. Carey Satterfield, Heewon Seo, Gregory A. Johnson, Guoyao Wu, Fuller W. Bazer

**Affiliations:** 1 Department of Animal Science, Texas A&M University, College Station, TX 77843-2471 USA; 2 Department of Veterinary Integrative Biosciences, Texas A&M University, College Station, TX 77843 USA

**Keywords:** Amino acids, Endometrium, Fructose, Gene expression, Glucose, Placenta, Polyamines, Progesterone

## Abstract

**Background:**

Administration of progesterone (P4) to ewes during the first 9 to 12 days of pregnancy accelerates blastocyst development by day 12 of pregnancy, likely due to P4-induced up-regulation of key genes in uterine epithelia responsible for secretion and transport of components of histotroph into the uterine lumen. This study determined if acceleration of blastocyst development induced by exogenous P4 during the pre-implantation period affects fetal-placental development on day 125 of pregnancy. Suffolk ewes (*n* = 35) were mated to fertile rams and assigned randomly to receive daily intramuscular injections of either corn oil vehicle (CO, *n* = 18) or 25 mg progesterone in CO (P4, *n* = 17) for the first 8 days of pregnancy. All ewes were hysterectomized on day 125 of pregnancy and: 1) fetal and placental weights and measurements were recorded; 2) endometrial and placental tissues were analyzed for the expression of candidate mRNAs involved in nutrient transport and arginine metabolism; and 3) maternal plasma, fetal plasma, allantoic fluid, and amniotic fluid were analyzed for amino acids, agmatine, polyamines, glucose, and fructose.

**Results:**

Treatment of ewes with exogenous P4 did not alter fetal or placental growth, but increased amounts of aspartate and arginine in allantoic fluid and amniotic fluid, respectively. Ewes that received exogenous P4 had greater expression of mRNAs for *SLC7A1*, *SLC7A2, SLC2A1*, *AGMAT*, and *ODC1* in endometria, as well as *SLC1A4, SLC2A5, SLC2A8* and *ODC1* in placentomes. In addition, AZIN2 protein was immunolocalized to uterine luminal and glandular epithelia in P4-treated ewes, whereas AZIN2 localized only to uterine luminal epithelia in CO-treated ewes.

**Conclusions:**

This study revealed that exogenous P4 administered in early pregnancy influenced expression of selected genes for nutrient transporters and the expression of a protein involved in polyamine synthesis on day 125 of pregnancy, suggesting a ‘programming’ effect of P4 on gene expression that affected the composition of nutrients in fetal-placental fluids.

**Supplementary Information:**

The online version contains supplementary material available at 10.1186/s40104-021-00567-1.

## Background

In sheep, the uterine luminal (LE), superficial glandular (sGE) and glandular (GE) epithelia are secretory cells that secrete and transport water, amino acids, hexose sugars, ions, growth factors, hormones, enzymes, cytokines, mitogens, and vitamins (collectively referred to as histotroph) into the uterine lumen early in pregnancy [1, 2]. Histotroph is also transported into the fetal-placental vasculature and accumulates in allantoic and amniotic fluids to support growth and development of the conceptus (embryo/fetus and associated placental membranes) [1].

Amino acids are important building blocks for synthesis of peptides and protein-based molecules, including cell signaling molecules and hormones. Amino acids themselves are important for fetal-placental growth, or can act in concert with polyamines to mediate embryogenesis, placental growth, and angiogenesis to increase utero-placental blood flow [3–5]. Polyamines (putrescine, spermidine, and spermine) and agmatine are products of amino acid metabolism (primarily arginine, ornithine, and methionine) involved in mammalian embryogenesis, angiogenesis, and functional aspects of trophectoderm cells [4, 6–8]. By binding nucleic acids and proteins, polyamines affect gene transcription, mRNA translation, and protein synthesis, thereby participating in cell growth, proliferation, communication, membrane trafficking, and motility [9–13].

In most eukaryotic cells, glucose is the primary substrate for the production of adenosine triphosphate (ATP), nicotinamide adenine dinucleotide phosphate hydrogen (NADPH),ribose [14]. Glucose is an important metabolic substrate for the conceptus, but fetal and placental tissues generally do not produce it themselves and thus must rely on glucose from the mother [15–17]. Interestingly, fructose is the most abundant hexose sugar in the fetal fluids and blood of ungulates and cetaceans and is considered ‘sequestered’ from the mother [18–20]. Fructose has multiple fates once inside a trophectoderm cell: it may enter the hexosamine biosynthesis pathway to produce glycosaminoglycans such as hyaluronic acid and UDP-N-acetylglucosamine, or its metabolic intermediates may be utilized to produce nucleic acids via one-carbon metabolism or NADPH and ribose sugars via the pentose phosphate pathway [21–23].

Molecules secreted as histotroph, the proteins that facilitate their transport, and the signaling mechanisms stimulated by these molecules are regulated in both a spatial (cell-specific) and temporal manner. In cattle, a delayed increase in circulating concentrations of progesterone (P4) reduces or delays secretion of interferon tau (IFNT), the pregnancy recognition signal in ruminants, and hinders conceptus development [24]. However, P4 supplementation after ovulation enhances bovine conceptus development and secretion of IFNT [25, 26]. This indicates that P4 regulates early conceptus growth by modifying the timing of gene expression by cells of the uterine endometrium and their secretory activity. However, it is clearly the timing of early increases in concentrations of circulating P4 in maternal plasma, rather than the final concentrations of circulating P4, that induces accelerated development of the conceptus [27].

In previous studies, P4 was administered to ewes 36 h after mating to observe effects on development of pre-implantation conceptuses [28–30]. In those studies, exogenous P4 administration advanced conceptus development by stimulating conceptus elongation earlier than conceptuses from control ewes. It was speculated that this was due to increased nutrient trafficking into the uterine lumen, as the results indicated increased expression of mRNAs for glucose and amino acid transporters in the uterine endometria of P4 treated ewes, as well as increases in recoverable nutrients, including glucose and arginine. Enhanced fetoplacental growth has been reported when ewes were administered P4 during the first 3 days of pregnancy [31, 32]. Those studies showed that conceptuses exposed to a P4-primed uterus had increased fetal crown-rump lengths and organ weights at day 76 of pregnancy, compared to fetuses from untreated ewes. However, little is known as to how these effects translate to the uterine environment and conceptus development in late gestation. The aim of this study was to determine if: 1) an exogenous systemic P4 treatment during the pre-implantation period of pregnancy has long-term effects on fetal and placental development, and 2) P4-induced modifications of uterine and placental gene expression influence the composition of uterine secretions, fetal fluids, and maternal and fetal plasma. Our hypothesis was that the P4-induced altered uterine environment that accelerates conceptus development during early pregnancy would result in advanced fetal-placental development due to alterations in expression of nutrient transporters and/or composition of fetal fluids in late gestation.

## Methods

### Animals

All experimental procedures followed the Guide for the Care and Use of Agriculture Animals in Research and Teaching and approved by the Institutional Animal Care and Use Committee of Texas A&M University. The sheep used in this study were reproductively mature, as determined by exhibiting estrous cycles of normal duration in the presence of a vasectomized ram. All animals had an average body condition score (BCS) of 4 (ranged 3–5) for the duration of the study. Ewes were housed in a covered barn in group pens with free access to water and fed a commercially available complete pelleted sheep ration at a rate to meet NRC requirements [33].

### Experimental design and tissue collection

Estrous cycles of mature Suffolk ewes (*Ovis aries*) were synchronized using a progesterone intravaginal insert (CIDR, Zoetis) for 12 days, and received an intramuscular injection of prostaglandin F_2α_ (20 mg Lutalyse, Zoetis) upon CIDR removal. Ewes were observed for estrus (designated as day 0) in the presence of a vasectomized ram. Upon detection of estrus (day 0) ewes were placed with a fertile ram for 36 h and rams were changed every 12 h during this period. A total of five rams used in this study, and all ewes were exposed to three of these rams for the duration of estrus. Ewes were assigned randomly to receive daily intramuscular injections of either corn oil and ethanol vehicle (CO; *n* = 18) or 25 mg progesterone dissolved in ethanol in corn oil vehicle (P4; *n* = 17) from day 1.5 (36 h after onset of estrus) through day 8 of pregnancy. On day 35 of gestation, ewes were subjected to transabdominal ultrasonography to determine pregnancy status. At day 125 of pregnancy, ewes were euthanized and hysterectomized after collecting blood in an EDTA coated tube via jugular venipuncture. Pregnancy type of each ewe was characterized by the number of fetuses (singleton vs twins) present at the time of hysterectomy. After the uteri were weighed, the chorioallantois was separated from the endometrium to expose the fetus and placental membranes. Samples of allantoic and amniotic fluids were collected, and volumes were determined. Allantoic and amniotic fluid were stored at −20 °C following centrifugation (10,000 × *g* for 10 min). Sections of inter-caruncular endometrium and whole placentomes were collected and either frozen in liquid nitrogen and stored at −80 °C or fixed in 4% paraformaldehyde and dehydrated in 70% ethanol for 48 h prior to embedding in Paraffin wax. Fetal blood was collected by cardiac puncture and transferred to an EDTA coated tube. After separating the fetus and placenta, placental length and weight was measured, and the number of placentomes was determined. The fetuses were also weighed, and measurements indicative of fetal growth (crown-rump length, abdominal circumference, and chest circumference) were taken.

### Radioimmunoassay analysis of progesterone

Maternal blood samples were collected immediately prior to euthanasia of ewes and stored on ice until processing. Plasma was collected following centrifugation (8000 × *g* for 7 min) and stored at −20 °C until analyzed. Concentrations of progesterone in maternal plasma were determined as previously described [34] using a Progesterone Coated Tube Radioimmunoassay Kit (07-270102, MP Diagnostics) validated with P4 in ovine plasma.

### Analysis of amino acids, agmatine, and polyamines

Concentrations of amino acids, polyamines, and agmatine were determined in allantoic fluid, amniotic fluid, maternal plasma, and fetal plasma by high performance liquid chromatography (HPLC) using modified procedures described previously [35–37]. Briefly, 100 μL of sample was acidified with 100 μL of 1.5 mol/L HClO_4_ and neutralized with 50 μL 2 mol/L K_2_CO_3_. The neutralized supernatant was diluted as needed and used for analysis by an HPLC method involving precolumn derivatization *o*-phthaldialdehyde (OPA) reagent I or II. OPA reagent I (for quantification of polyamines and agmatine) was prepared by dissolving 50 mg of OPA (Sigma-Aldrich) and 50 mg N-acetyl-cysteine (Sigma-Aldrich) in 1.25 mL of HPLC-grade methanol (Fisher Scientific) followed by 11.2 mL 0.04 mol/L sodium borate (pH 9.5), and 0.5 mL of Brij-23 (Sigma-Aldrich) OPA reagent II (for amino acids) was prepared by dissolving 50 mg OPA in 1.25 mL HPLC-grade methanol, followed by 11.2 mL sodium borate (pH 9.5), 50 μL 2-mercaptoethanol, and 0.5 mL of Brij-23 (Sigma-Aldrich). The assay mixture contained 1.4 mL of HPLC-grade water (Fisher Scientific), 100 μL of 1.2% benzoic acid (in 40 mmol/L sodium borate, pH 9.5), and 100 μL of sample. The assay mixture was derivatized in an autosampler (model 712 WISP, Waters) with 30 mmol/L OPA reagent I or II, and 15 μL of the derivatized mixture was injected into a Supelco 3-μm-reversed-phase C18 column (150 mm × 4.6 mm inner diameter, Sigma-Aldrich). Amino acids, polyamines, and agmatine were separated using a solvent gradient comprised of solution A (0.1 mol/L sodium acetate, 18% methanol, and 1% tetrahydrofuran, pH 7.2) and solution B (methanol). Amino acids, polyamines, and agmatine in the samples were quantified relative to authentic standards using Millenium-32 Software (Waters). Total amounts of amino acids, polyamines, and agmatine in allantoic and amniotic fluid were calculated by multiplying the concentration by the fluid volume.

### Quantification of glucose and fructose

Allantoic and amniotic fluid, as well as fetal and maternal plasma, were analyzed for concentrations of glucose using a Glucose Assay Kit (STA-680, Cell Biolabs, Inc), as per the manufacturer’s instructions. Each fluid type was assayed on one plate, and each plate was read on a spectrophotometric plate reader (Spectramax M2, Molecular Devices) (nm = 540 nm) within 5 min after incubation. Kit standards were used to generate a standard curve from 0 to 100 μmol/L, and maternal plasma, fetal plasma, allantoic fluid, and amniotic fluid, were diluted (1:100, 1:80, 1:5, and 1:2, respectively) with 1× assay buffer to ensure sample concentrations were within the limits of the standard curve The limit of detection of the assay was 6.25 μmol/L. The intra and inter assay coefficients of variation were 2.5% and 11.5%, respectively. Allantoic and amniotic fluid data are expressed as total glucose [volume of fluid (mL) × concentration of glucose (μmol/L)] in the respective fluids.

Maternal plasma, fetal plasma, allantoic fluid, and amniotic fluid were analyzed for concentrations of fructose using a Fructose Assay Kit (EFRU-100; BioAssay Systems), as per manufacturer’s instructions. Each fluid type was assayed on one plate, and each plate was read immediately on a spectrophotometric plate reader (nm = 565). Kit standards were used to generate a standard curve from 0 to 1000 μmol/L, and allantoic fluid, amniotic fluid, and fetal plasma were diluted (1:8, 1:15, and 1:10, respectively) with double distilled water to ensure sample concentrations were within the limits of the standard curve. The limit of detection of the assay was 12 μmol/L. The intra and inter assay coefficients of variation were 5.4% and 9.5%, respectively. Allantoic and amniotic fluid data are expressed as total fructose [volume of fluid (mL) × concentration of fructose μmol/L] in the respective fluids.

### RNA extraction, cDNA synthesis, and quantitative real-time PCR analysis

RNA was extracted from ovine endometria and placentomes using Trizol (Invitrogen) as per manufacturer’s instructions and treated with RNase-Free DNase (Qiagen). The RNA was further purified using the RNeasy Mini Kit (Qiagen) as per manufacturer’s instructions. The RNA was quantified using a NanoDrop (ND-1000 Spectrophotometer), and the quality was assessed by electrophoresis (2100 Bioanalyzer, Agilent Technologies). Only samples with an RNA integrity number (RIN) greater than 7 were used. First-strand cDNAs were synthesized from 5 μg of total RNA using oligo (deoxythymidine) primers and SuperScript II Reverse Transcriptase (Invitrogen) as per the manufacturer’s instructions. Negative controls without reverse transcriptase were included to verify a lack of genomic contamination.

**Fig. 1:**
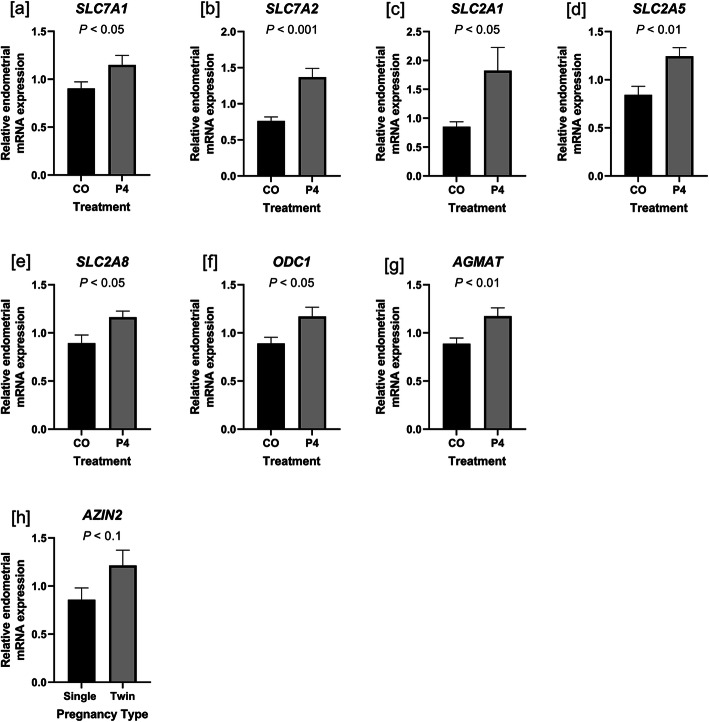
Endometrial expression of mRNAs affected by treatment (**a-g**) and fetal number (**h**) in ewes treated with either progesterone (P4) or corn oil (CO). Expression of mRNAs for *SLC7A1* (**a**), *SLC7A2* (**b**), *SLC2A1* (**c**), *SLC2A5* (**d**), SLC2A8 (**e**), ODC1 (**f**), and AGMAT (**g**) was greater in endometria of ewes treated with P4 compared to ewes treated with CO. Expression of mRNAs for AZIN2 (**h**) was greater in endometria of ewes pregnant with two fetuses compared to those with one fetus. Mean values and SEM are presented. *n* = 5–9 samples per group

**Fig. 2:**
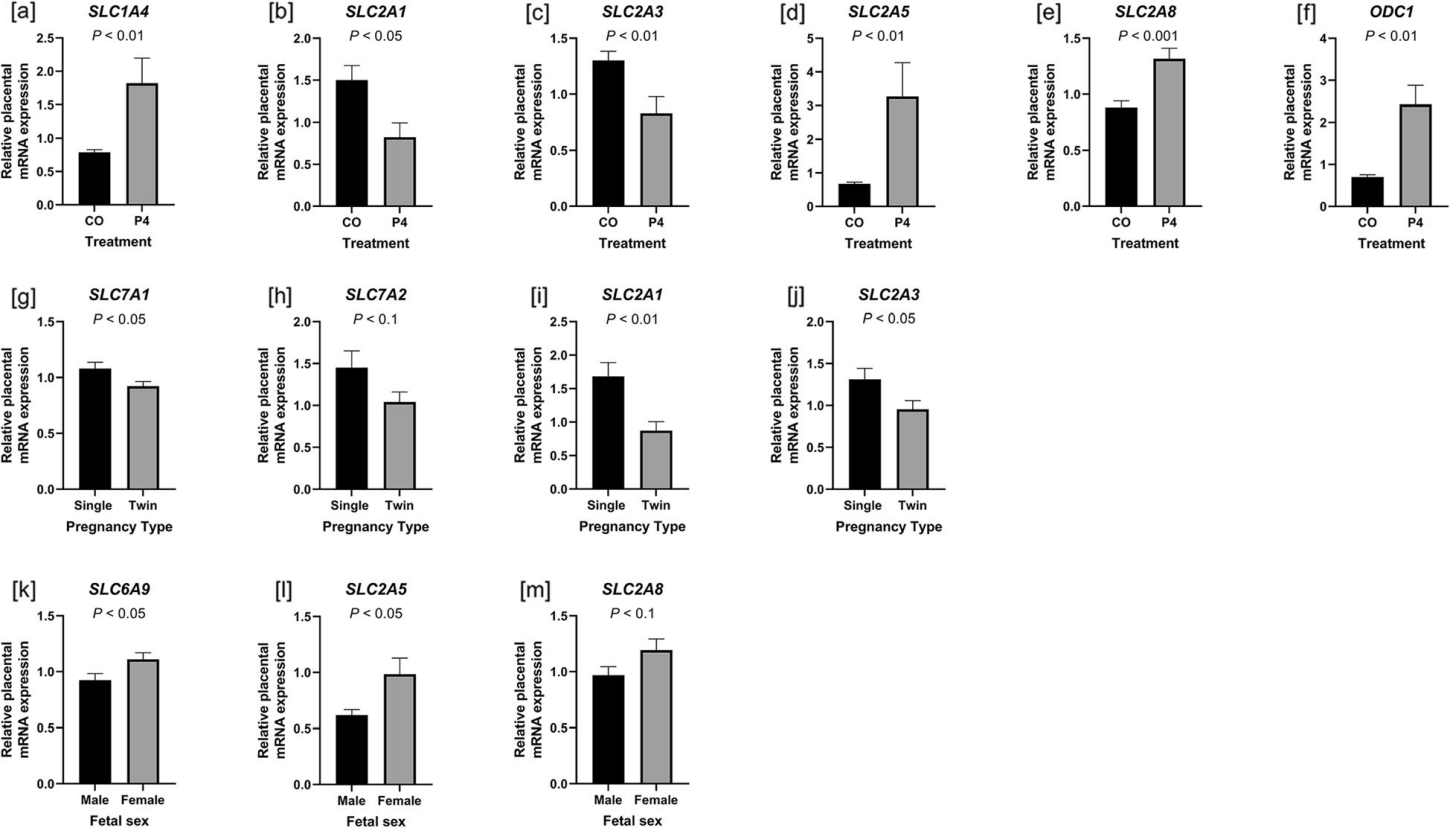
Placental expression of mRNAs affected by treatment (**a-f**), fetal number (**g-j**), and fetal sex (**k-m**) in ewes treated with either progesterone (P4) or corn oil (CO). Expression of mRNAs for *SLC1A4* (**a**), *SLC2A5* (**d**), *SLC2A8* (**e**), and *ODC1* (**f**) was greater in placentomes of ewes treated with P4 compared to those treated with CO, while *SLC2A1* (**b**) and *SLC2A3* (**c**) mRNA expression was lower in P4-treated ewes. Expression of mRNAs for *SLC7A1* (**g**), *SLC7A2* (**h**), *SLC2A1* (**i**), and *SLC2A3* (**j**) was greater in placentomes of pregnancies with one fetus than those with two fetuses. Expression of mRNAs for *SLC6A9* (**k**), *SLC2A5* (**l**), and *SLC2A8* (**m**) was greater in placentomes of placentae associated with female compared to male fetuses. Mean values and SEM are presented. *n* = 5–9 samples per group

Quantitative polymerase chain reaction (qPCR) was performed using the ABI prism 7900HT system (Applied Biosystems, Foster City, CA, United States) with Power SYBR Green PCR Master Mix (Applied Biosystems) as specified by the manufacturer to determine the levels of expression of mRNAs encoding for genes of interest. Primer sequences are listed in Supplementary Table 1. Primer efficiency and specificity were tested by generating a standard curve from pooled cDNA and by the inclusion of a dissociation curve for the qPCR reaction, respectively. Serial dilutions of pooled cDNA in nuclease-free water ranging from 1:2 to 1:256 were used as standards. All primer sets used amplified a single product (i.e. a dissociation curve with a single peak) and had an efficiency of between 95% and 105%. Each well contained 10% diluted cDNA, 30% nuclease-free water, 10% primer, and 50% SYBR Green reaction mix in a 10-μL reaction volume. Each sample was run in triplicate using the following conditions: 50 °C for 2 min, 95 °C for 10 min, followed by 40 cycles of 95 °C for 15 s and 60 °C for 1 min. All reactions were performed at an annealing temperature of 60 °C. For primers of interest with lower expression (i.e. Cq values above 30; *AZIN2* and *AGMAT*), 1 μL of cDNA was used in a modified pre-amplification step [38] using a Thermocycler (Eppendorf AG). Briefly, cDNA, nuclease-free water, forward and reverse primer, and SYBR were combined as described above in a 20-μL volume. The reaction was performed with the following conditions for 15 cycles: 94 °C for 30 s, 58 °C for 30 s, and 72 °C for 30 s. The reference genes *SDHA* and *GAPDH* were determined to have stable expression in endometria and placentomes, respectively, by testing for effects of treatment, pregnancy type, fetal sex, and their combinations. *P* values > 0.1 indicated that expression of these genes was unaffected, and thus were used to normalize expression of mRNAs of interest. The abundance of mRNAs was quantified using the ΔΔCq method, and these values are represented in Figs. 1 and 2 [28].

### Localization of proteins

Immunohistochemical localization of SLC2A5, ODC1, and AZIN2 proteins in endometria, and AZIN2 and AGMAT proteins in placentomes, was performed using a Vectastain ABC Universal Kit (Vector Laboratories, PK-6200) as per manufacturer’s instructions. Antibody information is listed in Supplementary Table 2. Briefly, sections of endometria and placentomes were cut (5 μm) and rehydrated through CitriSolv (Decon Labs, Inc.) and decreasing concentrations of ethanol to water. For SLC2A5, ODC1, and AGMAT proteins, antigen retrieval was performed using boiling 0.1 mol/L sodium citrate buffer (pH 6). For AZIN2, slides were incubated for 8 min at 37 °C in a solution of 0.5 mg/mL Protease (Sigma-Aldrich, P5147) dissolved in 1× PBS (phosphate buffered saline) for antigen retrieval. Endogenous peroxide activity was blocked by incubating slides in 0.3% hydrogen peroxide (Sigma Aldrich) in methanol at room temperature for 15 min. Sections were incubated for 1 h at room temperature in normal horse serum (Vectastain ABC Universal Kit) to block non-specific binding sites. Sections were incubated in primary antibody in a humidified chamber for SLC2A5 (Sigma-Aldrich, AV42096; 1:100 dilution), ODC1 (Abcam, ab97395; 1:450 dilution), and AZIN2 (Abcam, ab192771; 1:350 dilution) in endometria, and AZIN2 (1:250 dilution) and AGMAT (Abcam, ab231894; 1:100 dilution) in placentomes. Rabbit immunoglobulin G (IgG) (Vector Laboratories) was used as a negative control at the same concentrations for each antibody used. After overnight incubation at 4 °C, slides were washed in PBS and incubated in a humidified chamber with biotinylated anti-rabbit IgG secondary antibody (Vectastain Elite ABC Kit; 1:200 dilution in PBS with 1.5% normal horse serum) at 37 °C for 1 h. Slides were then incubated at 37 °C for 30 min in a humidified chamber with Vectastain Elite ABC Reagent (Vectastain Elite ABC Kit). Immunoreactive protein was visualized using 3,3′-diaminobenzidine tetrahydrochloride (Sigma-Aldrich). Sections were counterstained with hematoxylin and dehydrated through increasing concentrations of ethanol to CitriSolv before affixing coverslips with Permount mounting medium (Fisher Scientific). Images were captured of representative fields using a Nikon Eclipse Ni microscope and NIS Software (Nikon).

### Image analysis

AZIN2-stained endometrial stromal images taken at 10× magnification containing uterine glands were analyzed using ImageJ. Regions of the stroma containing uterine glands were analyzed in the same image. Images were split into red, green, and blue channels. Using the free-hand drawing tool, the percentage staining per uterine gland was quantified using the blue channel at a threshold of 100-pixel intensity.

### Statistical analysis

Statistical analyses were performed using either SAS (Version 9.4) or Genstat. Normality of the distribution of data was assessed, and a *P* value of ≥0.05 indicated that the data were not normally distributed. The ROUT test for outliers was performed to identify data points for exclusion. Normality was reassessed after exclusion of the outliers. Transformations were carried out if necessary to achieve a Gaussian distribution. ANOVA with a post-hoc Tukey test was performed on data with a normal distribution. If data were not normally distributed, Kruskal-Wallis and Mann Whitney tests were performed.

## Results

A summary of measurements affected by progesterone (P4) treatment, pregnancy type, and fetal sex may be found in Supplementary Table 3.

### Comparison of pregnancy rates, pregnancy type, and fetal sex

To determine whether P4 treatment influenced pregnancy rates, the number of pregnancies were compared between the P4- and CO- treated groups at day 35 of gestation. P4-treated ewes had a significantly lower pregnancy rate (64.7%) compared to CO-treated ewes (94.4%) (*P* < 0.05). At necropsy, there was no significant difference in pregnancy rate between CO- (*n* = 14) and P4- (*n* = 10) treated ewes (*P* > 0.05). There were 10 total ewes pregnant with two fetuses (5 CO- and 5 P4-treated ewes), and 14 were pregnant with singletons (9 CO- and 5 P4-treated ewes) at necropsy. There were a total of 21 male fetuses and 13 female fetuses (12 males and 7 females from CO-treated ewes, and 9 males and 6 females from P4-treated ewes). There were no differences in pregnancy type (singleton vs. twins) or distribution of fetal sex due to treatment (*P* > 0.05).

### Concentrations of progesterone in maternal plasma

There was no difference (*P* > 0.05) in concentrations of progesterone in maternal plasma between P4-treated (22.4 ± 2.4 ng/mL) and CO-treated (21.5 ± 1.6 ng/mL) ewes on day 125 of pregnancy. An increase in systemic P4 for ewes treated with P4 was confirmed on day 9 of pregnancy (Hoskins et al., unpublished results).

### Fetal and placental phenotypes

Measurements of fetal development, including weight, crown-rump length, abdominal circumference, and chest circumference are summarized in Table 1. Fetal weight, crown-rump length, abdominal circumference, chest circumference, as well as placental weight, placental length, and number of placentomes were not different between treatment groups (*P* > 0.05). Male fetuses were heavier (*P* < 0.05) and had greater chest circumferences (*P* < 0.05) than female fetuses. Placental weight, placental length, number of placentomes, and volumes of allantoic and amniotic fluid were also measured as indicators of placental development (Table 1). Placental length (*P* < 0.01) and number of placentomes per placenta (*P* < 0.01) were greater in pregnancies with a singleton fetus than twin-fetuses. Volumes of allantoic fluid and amniotic fluid were not different due to P4 treatment, fetal number, or their interaction (*P* > 0.05).

**Table 1:** Parameters of growth for fetuses and placentae

Measurement	Effect					
	Treatment		Pregnancy type		Fetal sex	
	CO	P4	Single	Twin	Male	Female
Fetal weight, g	3648 ± 174.1	3776 ± 157.1	**4089 ± 181.7**^**a**^	**3435 ± 127.2**^**b**^	**3916 ± 137**^**a**^	**3470 ± 98.7**^**b**^
Fetal CRL, cm	53.1 ± 0.8	52.9 ± 1	54 ± 1	52.3 ± 0.6	53.4 ± 0.8	52.3 ± 0.9
Fetal ab. circ., cm	33.7 ± 0.7	33.1 ± 0.8	34.3 ± 0.8	32.9 ± 0.7	33.91 ± 0.6	32.6 ± 0.9
Fetal chest circ., cm	32.6 ± 0.6	33.1 ± 0.5	**33.9 ± 0.5**^**a**^	**32.1 ± 0.5**^**b**^	**33.4 ± 0.4**^**a**^	**31.7 ± 0.7**^**b**^
Placenta weight, g	602.3 ± 35.4	522.7 ± 44.7	630.7 ± 41.2	522.8 ± 36.2	503.7 ± 43.4	601.9 ± 35.4
Placental length, cm	100.6 ± 4.7	99.4 ± 6.7	**116.7 ± 4.9**^**a**^	**87.3 ± 3.4**^**b**^	92.4 ± 4.7	103.7 ± 5
Number of cotyledons	54 ± 3.7	50.6 ± 5.2	**67 ± 3.6**^**a**^	**42.5 ± 2.7**^**b**^	47.2 ± 4.3	55.6 ± 3.9
Allantoic fluid volume, mL	617.2 ± 56.1	651.9 ± 58.7	662.1 ± 68.3	609.7 ± 49.6	564.7 ± 81.7	665.6 ± 44.2
Amniotic fluid volume, mL	598.8 ± 47.6	665.9 ± 87.1	660.4 ± 55.8	602.6 ± 66.5	688.8 ± 87.5	592.4 ± 50.8

Measurements of fetuses and placentae from ewes treated with either CO (*n* = 14) or P4 (*n* = 10), singleton pregnancies (*n* = 14) or twin pregnancies (*n* = 10), and male fetuses (*n* = 21) or female fetuses (*n* = 13). Values represent means ± SEM. Different superscripts within Effect columns are significantly different (*P* < 0.05)

### Amino acids in maternal and fetal plasma, allantoic fluid, and amniotic fluid

Concentrations of amino acids in maternal and fetal plasma are summarized in Tables 2 and 3, respectively, and Supplementary Fig. 1. The most abundant amino acids in maternal plasma were glutamate, glycine, and alanine. There were no differences in concentrations of amino acids in maternal plasma between P4- and CO-treated ewes (*P* > 0.05). Concentrations of aspartate, threonine, arginine, valine, and ornithine were greater (*P* < 0.05) in plasma of ewes with a singleton fetus compared to those for ewes with twin fetuses. Similarly, there was a tendency for greater concentrations of citrulline in plasma of ewes with a single fetus (*P* < 0.1). Interestingly, concentrations of glutamate in maternal plasma were less (*P* < 0.05) for ewes with a singleton fetus compared to twin fetuses. Arginine was more abundant in maternal plasma of P4-treated ewes carrying twins compared to P4-treated ewes carrying a singleton (*P* < 0.05).

**Table 2:** Concentrations of amino acids, agmatine, polyamines (nmol/mL), and hexose sugars (μmol/L) in maternal plasma

Nutrient	Effect			
	Treatment		Pregnancy type	
	CO	P4	Single	Twin
*Amino acid*				
Alanine	176.9 ± 16	173.4 ± 11.1	172.1 ± 11.1	179.8 ± 19.5
β-Alanine	10.9 ± 1.3	10.2 ± 0.6	10.7 ± 1.2	10.4 ± 1
Arginine	118 ± 8.4	121.7 ± 11.3	**134.7 ± 8.2**^**a**^	**100.6 ± 7.9**^**b**^
Asparagine	24 ± 2.5	22.6 ± 1.8	21.5 ± 2.2	25.8 ± 2.3
Aspartate	1.0 ± 0.2	0.84 ± 0.2	**1.2 ± 0.2**^**a**^	**0.67 ± 0.2**^**b**^
Citrulline	96 ± 14.1	102.7 ± 10.3	112.6 ± 15.1	81 ± 6.1
Glutamate	202.1 ± 14.7	213.6 ± 26.1	**181.1 ± 12.1**^**a**^	**239.3 ± 23.4**^**b**^
Glutamine	56.6 ± 6.9	50.5 ± 11.1	50.3 ± 8.3	59.1 ± 8.7
Glycine	428.2 ± 28.2	440.4 ± 25	431.4 ± 25	435.2 ± 28.8
Histidine	23.5 ± 1.8	22.2 ± 1.8	22.4 ± 1.5	23.7 ± 1.9
Isoleucine	74.1 ± 7.3	66.3 ± 5.4	75.6 ± 6.3	65.1 ± 7.6
Leucine	114.9 ± 10.8	94.5 ± 5.6	115.1 ± 8.4	96.2 ± 11.6
Lysine	71.2 ± 9	68.2 ± 9.8	75.1 ± 9.3	63.5 ± 9
Methionine	20 ± 1.8	20.7 ± 1.3	20.7 ± 1.4	19.8 ± 2.1
Ornithine	23.6 ± 3.7	23.7 ± 3.6	**28.6 ± 3.7**^**a**^	**17.2 ± 2.7**^**b**^
Phenylalanine	36.7 ± 3.2	34.8 ± 1.5	37.1 ± 1.9	37.2 ± 3.9
Serine	66.7 ± 7.8	58.3 ± 4.8	57.6 ± 7.1	70.6 ± 6.7
Taurine	60.3 ± 5.3	61.6 ± 5.9	61.4 ± 5.1	60.2 ± 6.2
Threonine	68.1 ± 9	54.6 ± 5.2	**75.1 ± 7.2**^**a**^	**46.9 ± 7.5**^**b**^
Tryptophan	22.4 ± 1.9	22 ± 1.7	23.2 ± 1.4	21.1 ± 2.4
Tyrosine	49.3 ± 4.3	49 ± 3.6	53.1 ± 3.4	44.2 ± 4.8
Valine	138.9 ± 14.3	119.1 ± 10.7	**148.2 ± 10.3**^**a**^	**109.1 ± 15.5**^**b**^
*Agmatine and polyamines*				
Agmatine	130.3 ± 13.4	166.1 ± 18.3	155.6 ± 15.4	130.5 ± 6.1
Putrescine	2.1 ± 0.6	2.3 ± 0.8	2.9 ± 0.8	1.3 ± 0.3
Spermidine	1.7 ± 0.3	1.7 ± 0.4	2.1 ± 0.3	1.2 ± 0.4
Spermine	0.73 ± 0.2	0.45 ± 0.1	0.76 ± 0.2	0.44 ± 0.1
*Hexose sugars*				
Glucose	1485 ± 162.9	1617 ± 110.7	1691 ± 137.9	1296 ± 144.2
Fructose	331.5 ± 33.8	303.2 ± 39.8	324.3 ± 32.9	313.2 ± 41.7

Concentrations of nutrients in maternal plasma from ewes treated with either CO (*n* = 14) or P4 (*n* = 10) that carried either singleton (*n* = 14) or twin pregnancies (*n* = 10). Values represent means ± SEM. Different superscripts within Effect columns are significantly different (*P* < 0.05)

**Table 3:** Concentrations of amino acids, agmatine, polyamines (nmol/mL), and hexose sugars (μmol/L) in fetal plasma

Nutrient	Effect					
	Treatment		Pregnancy type		Fetal sex	
	CO	P4	Single	Twin	Male	Female
*Amino acid*						
Alanine	512.8 ± 36.7	492.3 ± 55.6	**456 ± 38.6**^**a**^	**545.8 ± 46.6**^**b**^	500.5 ± 40.5	509 ± 51.6
β-Alanine	129.6 ± 12.6	121 ± 15.5	144.5 ± 16.5	109.5 ± 9.8	126.8 ± 9.8	124.5 ± 19.9
Arginine	137.2 ± 14.7	138.4 ± 10.6	135.6 ± 11.1	139.6 ± 14.9	144.6 ± 13.1	127 ± 12.6
Asparagine	59.4 ± 6.2	54.3 ± 4.8	54 ± 4.7	60 ± 6.5	60 ± 6.2	53 ± 4
Aspartate	33.1 ± 4.1	32.6 ± 5.4	**25.4 ± 3**^**a**^	**39.4 ± 5**^**b**^	31.6 ± 4.4	34.7 ± 5
Citrulline	87.3 ± 6.1	93.7 ± 11.7	98.9 ± 9.2	82.3 ± 7.8	87.3 ± 5.9	94.1 ± 12.6
Glutamate	600.5 ± 58.5	549.9 ± 78.2	507.4 ± 62.9	640.9 ± 66.2	578.5 ± 62.4	578.8 ± 73.5
Glutamine	232.7 ± 19.1	245.9 ± 25.1	218.5 ± 20.5	255.8 ± 21.5	242.6 ± 19.1	232.2 ± 25.5
Glycine	556.6 ± 56.7	622.6 ± 68.4	**495.5 ± 36.3**^**a**^	**663.6 ± 70.3**^**b**^	566.2 ± 55.1	613.6 ± 72.3
Histidine	49.7 ± 3	55.4 ± 5.3	54.2 ± 4.7	50.4 ± 3.5	54.2 ± 3.8	49.1 ± 4.2
Isoleucine	98 ± 5.6	89.3 ± 8.4	92.8 ± 6.9	95.5 ± 6.9	95.2 ± 6.3	92.8 ± 7.7
Leucine	198.1 ± 9.9	179.6 ± 17.5	187.2 ± 14.5	192.5 ± 12.7	186.6 ± 13.1	196.1 ± 12.9
Lysine	116.9 ± 9.5	112.5 ± 14.9	124.3 ± 13.8	106.9 ± 9.7	120.9 ± 11.4	106.1 ± 11.6
Methionine	26.8 ± 2.3	28.1 ± 3.3	**31.5 ± 3.2**^**a**^	**23.7 ± 1.9**^**b**^	28.5 ± 2.7	25.6 ± 2.6
Ornithine	123.6 ± 9.8	127.7 ± 19.9	136.6 ± 16.4	115.5 ± 12.4	125.1 ± 12.8	125.8 ± 17.1
Phenylalanine	104.2 ± 6.1	92.6 ± 7.6	98.9 ± 7.7	99.4 ± 6.3	99.7 ± 6.4	98.4 ± 7.6
Serine	860.5 ± 72.1	752.5 ± 76.3	**697.8 ± 53.7**^**a**^	**915 ± 79.9**^**b**^	843.7 ± 64.7	768.7 ± 90.6
Taurine	128.7 ± 13.5	109.4 ± 16.1	134.1 ± 18	108.3 ± 11	123.5 ± 12.2	115.6 ± 18.8
Threonine	239.9 ± 24.8	205.4 ± 26.7	**277.1 ± 30.7**^**a**^	**179.4 ± 13.5**^**b**^	**256 ± 25.5**^**a**^	**178.4 ± 18.5**^**b**^
Tryptophan	35.4 ± 2.3	31.1 ± 2.8	**36.9 ± 3**^**a**^	**30.6 ± 1.9**^**b**^	34.6 ± 2.6	31.9 ± 2.4
Tyrosine	115.5 ± 10.8	100.1 ± 8.9	116.8 ± 13.1	101.9 ± 7.3	103 ± 9.1	117.6 ± 12.1
Valine	256.8 ± 13.8	218.6 ± 19.3	**262.3 ± 18**^**a**^	**220.3 ± 14.3**^**b**^	244.6 ± 15.7	233.7 ± 18.4
*Agmatine and polyamines*						
Agmatine	214.5 ± 8.5	231.9 ± 13.7	226.7 ± 9.6	218 ± 11.9	225.4 ± 11.5	219.9 ± 10.5
Putrescine	8.0 ± 0.8	6.5 ± 1.1	**8.8 ± 0.9**^**a**^	**6.1 ± 0.7**^**b**^	7.9 ± 0.9	6.6 ± 1
Spermidine	2.0 ± 0.3	2.0 ± 0.4	2.5 ± 0.4	1.5 ± 0.2	2.1 ± 0.3	1.8 ± 0.3
Spermine	7.3 ± 0.9	6.3 ± 1.1	7.3 ± 1	6.4 ± 1	7.3 ± 1.1	6.1 ± 0.6
*Hexose sugars*						
Glucose	1934 ± 212.8	2231 ± 475.4	1722 ± 237.7	2369 ± 374.6	2377 ± 301.4	1532 ± 303
Fructose	2019 ± 640.9	3212 ± 1166	3836 ± 1260	1437 ± 225	1973 ± 640	3277 ± 1160

Concentrations of nutrients in fetal plasma out of ewes treated with either CO (*n* = 14) or P4 (*n* = 10), from either singleton (*n* = 14) or twin pregnancies (*n* = 10), and either male (*n* = 21) or female (*n* = 13). Values represent means ± SEM. Different superscripts within Effect columns are significantly different (*P* < 0.05)

Overall, glutamate, serine, glycine, and alanine were most abundant in fetal plasma. There were no differences in concentrations of amino acids in fetal plasma from P4- or CO-treated ewes (*P* > 0.05). Concentrations of aspartate (*P* < 0.05), serine (*P* < 0.05), and glycine (*P* < 0.1) were greater for fetuses from twin compared to singleton fetus pregnancies. Conversely, concentrations of threonine (*P* < 0.05), β-alanine (*P* < 0.1), tryptophan (*P* < 0.1), methionine (*P* < 0.05), and valine (*P* < 0.1) were lower in plasma of fetuses from twin pregnancies than fetuses from singleton pregnancies. Threonine was the only amino acid in greater concentrations in plasma of male fetuses (*P* < 0.05). A comparison of maternal and fetal plasma indicated greater concentrations of all amino acids, except for arginine and citrulline, in fetal plasma than maternal plasma (*P* < 0.001).

Total amounts of amino acids in allantoic and amniotic fluids, calculated by multiplying the concentration by the volume of the respective placental fluid, are summarized in Tables 4 and 5, respectively, and Supplementary Fig. 1. There was less glutamate in allantoic fluid of P4-treated than CO-treated ewes (*P* < 0.05), while allantoic fluid of P4-treated ewes tended to have greater amounts of aspartate (*P* < 0.1) than that for CO-treated ewes. Allantoic fluid from singleton pregnancies had more taurine than allantoic fluid from twin pregnancies (*P* < 0.05). There was more serine, glutamine, histidine, glycine, threonine, arginine, taurine, tryptophan, phenylalanine, and isoleucine in allantoic fluid associated with male than female fetuses (*P* < 0.05), and total amounts of leucine also tended to be greater in allantoic fluid associated with male fetuses (*P* < 0.1). Allantoic fluid associated with male fetuses of P4-treated ewes had significantly (*P* < 0.05) greater abundances of arginine, histidine, glutamine, glycine, and threonine, with a tendency (*P* < 0.1) for greater amounts of aspartate, tryptophan, taurine, and leucine compared to allantoic fluid associated with female fetuses of P4-treated ewes.

**Table 4:** Total amounts (concentration × volume) of amino acids, agmatine, polyamines, and hexose sugars (mg) in allantoic fluid

Nutrient	Effect					
	Treatment		Litter size		Fetal sex	
	CO	P4	Single	Twin	Male	Female
*Amino acid*						
Alanine	39 ± 6.1	30.9 ± 9	40.8 ± 7.4	31.3 ± 7.3	40.9 ± 6.5	25.6 ± 8.5
β-Alanine	51.2 ± 12.2	36.4 ± 9.8	39.2 ± 14.7	48.6 ± 9.3	45.3 ± 8.1	43.6 ± 17.9
Arginine	57.4 ± 9.8	64.2 ± 20.6	46.4 ± 9.3	70.1 ± 16.4	**75.2 ± 14.4**^**a**^	**33.2 ± 10.1**^**b**^
Asparagine	11 ± 3.7	7.3 ± 2	9.2 ± 2	9.5 ± 3.6	**11.48 ± 3.3**^**a**^	**5.4 ± 1.6**^**b**^
Aspartate	4 ± 0.6	5.7 ± 0.8	4.7 ± 0.8	4.8 ± 0.6	5.3 ± 0.6	3.8 ± 0.7
Citrulline	16.3 ± 3.9	8.7 ± 2.2	14.9 ± 2.8	11.5 ± 3.7	15.7 ± 3.2	7.8 **±** 3.2
Glutamate	**15.2 ± 1.6**^**a**^	**10.8 ± 1.2**^**b**^	13.5 ± 1.5	13.1 ± 1.5	14.3 ± 1.1	11.4 ± 2.2
Glutamine	54.5 ± 10.3	58.1 ± 15.6	70.1 ± 12.4	45.4 ± 12.2	**71.3 ± 11.8**^**a**^	**29.7 ± 9.8**^**b**^
Glycine	27.4 ± 5	29.8 ± 6.8	30.8 ± 6.2	26.8 ± 5.4	**35 ± 5.1**^**a**^	**16.4 ± 5.2**^**b**^
Histidine	19.8 ± 5.9	15.4 ± 3.6	19.5 ± 3.1	16.7 ± 5.9	**18.6 ± 2.7**^**a**^	**8.2 ± 2.4**^**b**^
Isoleucine	5.6 ± 1.9	3.4 ± 0.9	5.1 ± 0.9	4.3 ± 1.8	**5.8 ± 1.6**^**a**^	**2.4 ± 0.9**^**b**^
Leucine	12.2 ± 3.8	7.2 ± 1.7	10.4 ± 1.7	9.7 ± 3.7	9.3 ± 1.4	5.9 ± 2
Lysine	27.9 ± 3.9	31.2 ± 7.4	34.4 ± 6.2	35 ± 10.5	**41.8 ± 9.4**^**a**^	**21.9 ± 5.9**^**b**^
Methionine	4.1 ± 1.5	3.5 ± 1	3.3 ± 0.8	4.2 ± 1.4	3.4 ± 0.7	2.3 ± 0.7
Ornithine	11.51 ± 2.2	16.2 ± 5.4	17.8 ± 3.3	10.6 ± 3.8	14 ± 3.7	12.9 ± 3.5
Phenylalanine	6.4 ± 1.6	5 ± 1.1	6.9 ± 1	4.9 ± 1.6	**7.2 ± 1.4**^**a**^	**3.2 ± 0.9**^**b**^
Serine	1099 ± 143.3	998.4 ± 174.2	908.6 ± 147.4	1105 ± 157.6	**1219 ± 103.7**^**a**^	**667 ± 219.8**^**b**^
Taurine	417.8 ± 65.1	358.9 ± 58.7	**496.9 ± 81.9**^**a**^	**318.2 ± 43.5**^**b**^	**473.2 ± 58.7**^**a**^	**242.5 ± 38.8**^**b**^
Threonine	27 ± 7.3	24 ± 6.3	30.6 ± 7.9	22.2 ± 6.2	**33.8 ± 6.8**^**a**^	**10.8 ± 3.1**^**b**^
Tryptophan	7.6 ± 1.1	7.9 ± 1.4	9 ± 1.1	6.9 ± 1.2	**9.1 ± 1**^**a**^	**4.8 ± 1**^**b**^
Tyrosine	14 ± 4.4	9.1 ± 3.4	8.1 ± 1.3	14.4 ± 4.8	12.8 ± 3.6	10.1 ± 5.1
Valine	15.6 ± 3.7	9.5 ± 2.6	16.8 ± 3.2	10.2 ± 3.3	15.4 ± 3.2	8.2 ± 3.1
*Agmatine and polyamines*						
Agmatine	5.3 ± 0.7	5.8 ± 0.8	6.7 ± 0.9	4.6 ± 0.6	6.1 ± 0.6	4.3 ± 1.1
Putrescine	0.26 ± 0.04	0.23 ± 0.04	0.24 ± 0.03	0.25 ± 0.05	0.26 ± 0.02	0.22 ± 0.07
Spermidine	0.006 ± 0.01	0.06 ± 0.01	0.051 ± 0.01	0.076 ± 0.01	**0.086 ± 0.02**^**a**^	**0.057 ± 0.007**^**b**^
Spermine	0.11 ± 0.01	0.13 ± 0.02	0.12 ± 0.01	0.11 ± 0.02	0.11 ± 0.01	0.12 ± 0.02
*Hexose sugars*						
Glucose	11.9 ± 1.6	10.6 ± 1.0	12.4 ± 1.3	10.6 ± 1.4	11.6 ± 1	11 ± 2
Fructose	667.6 ± 144.3	688.7 ± 117.2	574.7 ± 87.6	748.4 ± 148.3	676.4 ± 93.6	677.7 ± 212.5

Total amounts of nutrients in allantoic fluid from ewes treated with either CO (*n* = 14) or P4 (*n* = 10), from either singleton (*n* = 14) or twin pregnancies (*n* = 10), and associated with either male (*n* = 21) or female (*n* = 13) fetuses. Values represent means ± SEM. Different superscripts within Effect columns are significantly different (*P* < 0.05)

**Table 5:** Total amounts (concentration × volume) of amino acids, agmatine, polyamines, and hexose sugars (mg) in amniotic fluid

Nutrient	Effect					
	Treatment		Litter size		Fetal sex	
	CO	P4	Single	Twin	Male	Female
*Amino acid*						
Alanine	4.5 ± 1	8.3 ± 2.3	5.9 ± 1.7	6.2 ± 1.5	5.5 ± 1.6	7.1 ± 1.4
β-Alanine	2.0 ± 0.9	1.5 ± 0.4	2.6 ± 1.2	1.2 ± 0.3	1.2 ± 0.3	2.7 ± 1.3
Arginine	**2.4 ± 0.6**^**a**^	**4.2 ± 0.8**^**b**^	3.7 ± 0.9	2.7 ± 0.6	2.8 ± 0.7	3.7 ± 0.8
Asparagine	15.4 ± 3.1	24.4 ± 5.8	23.8 ± 5.3	15.9 ± 3.6	17.1 ± 4	22.6 ± 4.8
Aspartate	0.60 ± 0.1	0.82 ± 0.2	0.83 ± 0.2	0.59 ± 0.1	0.57 ± 0.1	0.89 ± 0.2
Citrulline	1.8 ± 0.6	4.1 ± 1.2	2.7 ± 0.9	2.8 ± 0.9	2.2 ± 0.8	3.7 ± 1
Glutamate	6.4 ± 0.6	7.5 ± 1.4	7.1 ± 0.9	6.8 ± 1	6.7 ± 0.9	7.2 ± 1
Glutamine	6.9 ± 1.8	11.1 ± 3.1	10.1 ± 3.1	7.6 ± 1.9	7.3 ± 2.2	10.9 ± 2.7
Glycine	8.3 ± 0.6	12.2 ± 2.6	9 ± 1.1	10.6 ± 1.9	8.8 ± 1.4	11.7 ± 2.2
Histidine	1.3 ± 0.4	2.2 ± 0.6	2.2 ± 0.6	1.3 ± 0.4	1.5 ± 0.4	2 ± 0.5
Isoleucine	0.74 ± 0.3	1.0 ± 0.4	0.99 ± 0.5	0.78 ± 0.3	0.65 ± 0.3	1.2 ± 0.5
Leucine	3.2 ± 0.8	4.9 ± 1.5	4.6 ± 1.4	3.4 ± 0.9	3.6 ± 1	4.4 ± 1.3
Lysine	1.5 ± 0.4	2.8 ± 0.8	**3.2 ± 0.9**^**a**^	**1.2 ± 0.3**^**b**^	0.9 ± 0.7	2.3 ± 0.6
Methionine	0.74 ± 0.2	0.95 ± 0.2	0.82 ± 0.2	0.83 ± 0.2	0.59 ± 0.2	1.2 ± 0.3
Ornithine	0.34 ± 0.1	0.56 ± 0.2	0.59 ± 0.2	0.32 ± 0.1	0.33 ± 0.1	0.60 ± 0.2
Phenylalanine	0.70 ± 0.2	1.1 ± 0.5	0.68 ± 0.2	1.0 ± 0.4	0.81 ± 0.3	1.0 ± 0.3
Serine	36.3 ± 4.5	41.7 ± 5.7	37.2 ± 5.1	39.5 ± 4.9	38.2 ± 4.5	39.1 ± 5.8
Taurine	13.4 ± 3.2	8 ± 1	16 ± 4.1	7.6 ± 1.1	12.1 ± 2.4	9.5 ± 3.4
Threonine	2.8 ± 0.7	6.4 ± 2.1	5.8 ± 2	3.3 ± 0.9	4.9 ± 1.6	3.4 ± 0.9
Tryptophan	0.72 ± 0.1	0.86 ± 0.2	**1.0 ± 0.1**^**a**^	**0.61 ± 0.2**^**b**^	0.67 ± 0.2	0.95 ± 0.2
Tyrosine	1.7 ± 0.4	2.7 ± 0.7	2.1 ± 0.6	2.1 ± 0.6	1.9 ± 0.6	2.4 ± 0.6
Valine	1.9 ± 0.5	3.2 ± 1	2.3 ± 0.7	2.4 ± 0.8	2.2 ± 0.7	2.9 ± 0.7
*Agmatine and polyamines*						
Agmatine	0.14 ± 0.02	0.16 ± 0.05	0.17 ± 0.03	0.13 ± 0.04	0.15 ± 0.04	0.15 ± 0.03
Putrescine	0.021 ± 0.002	0.020 ± 0.004	0.028 ± 0.003	0.014 ± 0.002	0.022 ± 0.004	0.019 ± 0.003
Spermidine	0.12 ± 0.03	0.26 ± 0.07	0.17 ± 0.04	0.19 ± 0.05	**0.10 ± 0.02**^**a**^	**0.31 ± 0.07**^**b**^
Spermine	0.35 ± 0.1	0.15 ± 0.04	0.22 ± 0.07	0.30 ± 0.1	0.25 ± 0.07	0.29 ± 0.1
*Hexose sugars*						
Glucose	5.8 ± 0.9	5.1 ± 0.9	**6.9 ± 1**^**a**^	**4.4 ± 0.7**^**b**^	4.5 ± 0.4	7.2 ± 1.4
Fructose	721.3 ± 75.2	672 ± 178	768.6 ± 134.8	656.9 ± 106.1	666 ± 99.8	763 ± 149.4

Total amounts of nutrients in amniotic fluid from ewes treated with either CO (*n* = 14) or P4 (*n* = 10), from either singleton (*n* = 14) or twin pregnancies (*n* = 10), and associated with either male (*n* = 21) or female (*n* = 13) fetuses. Values represent means ± SEM. Different superscripts within Effect columns are significantly different (*P* < 0.05)

Total amounts of arginine were greater in amniotic fluid of P4-treated than CO-treated ewes (*P* < 0.05). Lysine (*P* < 0.05) and tryptophan (*P* < 0.1) were greater in amniotic fluid of singleton pregnancies compared to amniotic fluid of twin pregnancies. Ornithine tended to be present in greater amounts in amniotic fluid associated with female compared to male fetuses (*P* < 0.1).

### Agmatine and polyamines in maternal plasma, fetal plasma, allantoic fluid, and amniotic fluid

Concentrations of agmatine and polyamines in maternal and fetal plasma are summarized in Tables 2 and 3, respectively, and Supplementary Fig. 2. Overall, concentrations of agmatine in both fetal and maternal plasma were greater (*P* < 0.001) than spermidine, spermine, and putrescine. A comparison of fetal and maternal plasma revealed greater concentrations of agmatine, spermine, and putrescine in fetal plasma compared to maternal plasma (*P* < 0.001).

There were no differences in concentrations of agmatine or polyamines in maternal plasma due to P4 treatment (*P* > 0.05). Spermidine tended to be in greater abundance in plasma of ewes with a singleton fetus compared to ewes with twin fetuses (*P* < 0.1).

Putrescine tended to be more abundant in fetal plasma of CO-treated ewes with a twin pregnancy than P4-treated ewes with a twin pregnancy (*P* < 0.1), and greater concentrations of putrescine were detected in plasma of fetuses from singleton than twin pregnancies (*P* < 0.05). Spermine tended to be in greater abundance in fetal plasma from singleton than twin pregnancies, as well as in plasma from male fetuses from a single pregnancy than a male fetus from a twin pregnancy (*P* < 0.1).

Total amounts of polyamines in allantoic and amniotic fluid are summarized in Tables 4 and 5, respectively, and Supplementary Fig. 2. There were no differences in total amounts of polyamines in either allantoic or amniotic fluid due to P4 treatment (*P* > 0.05). In allantoic fluid, there was significantly more agmatine than spermidine, spermine, and putrescine (*P* < 0.001), and male fetuses tended to have greater amounts of agmatine in allantoic fluid than female fetuses (*P* < 0.1). Allantoic fluid associated with female fetuses of twin pregnancies had greater amounts of spermidine than female fetuses of singleton pregnancies (*P* < 0.05). Comparison of allantoic and amniotic fluids indicated that allantoic fluid had more agmatine than amniotic fluid (*P* < 0.001).

In amniotic fluid, putrescine was the least abundant polyamine (*P* < 0.05). Interestingly, agmatine tended to be in greater abundance in amniotic fluid of twin than singleton pregnancies. Spermidine was more abundant in amniotic fluid associated with male compared to female fetuses (*P* < 0.01). Amniotic fluid of P4-treated ewes had greater abundances of putrescine in singleton pregnancies compared to twin pregnancies (*P* < 0.05).

### Glucose and fructose in maternal and fetal plasma, allantoic fluid, and amniotic fluids

There were no differences in concentrations of glucose or fructose in maternal (Table 2) or fetal plasma (Table 3), nor any difference in total glucose or fructose in allantoic (Table 4) or amniotic fluid (Table 5) due to P4 treatment (*P* > 0.05). Concentrations of glucose and fructose in maternal and fetal plasma were not different due to fetal number or fetal sex (*P* > 0.05). Total amounts of glucose or fructose in allantoic fluid was not affected by fetal number or fetal sex (*P* > 0.05). Total amounts of glucose were greater in amniotic fluid associated with singleton pregnancies than twin pregnancies (*P* < 0.05), but was not different due to fetal sex (*P* > 0.05). Total amounts of fructose in amniotic fluid was not different due to fetal number or fetal sex (*P* > 0.05).

A comparison of glucose and fructose was performed (Supplementary Fig. 3). Concentrations of glucose were greater than fructose in maternal plasma, while concentrations of fructose were greater than glucose in fetal plasma (*P* < 0.001). In both allantoic and amniotic fluid, fructose was more abundant than glucose (*P* < 0.001).

### Expression of mRNAs in endometria and placentomes

Expression of mRNAs for the cationic amino acid transporters for arginine *SLC7A1* (*P* < 0.05) and *SLC7A2* (*P* < 0.001),glucose transporter *SLC2A1* (*P* < 0.05), fructose transporter *SLC2A5* (*P* < 0.01) ornithine decarboxylase (*ODC1*; *P* < 0.05), and agmatinase (AGMAT; *P* < 0.01) was greater in endometria from P4-treated compared to CO-treated ewes (Fig. 1a-g). Expression of *AZIN2* mRNA (also known as arginine decarboxylase [ADC]) tended to be greater in endometria from twin pregnancies than singleton pregnancies (*P* < 0.1) (Fig. 1h). Expression of *SLC6A9* (sodium- and chloride-dependent glycine transporter) and *SLC1A4* (sodium-dependent neutral amino acid transporter for alanine, serine, cysteine, threonine, and glutamate) mRNAs was not different in endometria from P4-treated and CO-treated (*P* > 0.1) (data not shown). In placentomes, expression of neutral amino acid transporter *SLC1A4* (*P* < 0.01), fructose transporter *SLC2A5* (*P* < 0.01), glucose and fructose transporter *SLC2A8* (*P* < 0.01), and *ODC1* mRNAs was greater in P4-treated compared to CO-treated ewes (*P* < 0.01) (Fig. 2a, d-f). Interestingly, expression of mRNAs for glucose transporters *SLC2A1* (*P* < 0.05) and *SLC2A3* (*P* < 0.01) was down-regulated in placentomes of P4-treated ewes (Fig. 2b and c). Expression of *SLC7A1* (*P* < 0.05), *SLC7A2* (*P* < 0.1), *SLC2A1* (*P* < 0.01), and *SLC2A3* (*P* < 0.05) mRNAs was greater in placentomes of pregnancies with a singleton fetus compared to pregnancies with twin fetuses (Fig. 2g-j). Intriguingly, expression of *SLC6A9* (*P* < 0.05), *SLC2A5* (*P* < 0.05), and *SLC2A8* (*P* < 0.1) mRNA was greater in placentomes associated with female compared male fetuses (Fig. 2k-m). Expression of AZIN2 mRNA in endometria was unaffected by either P4 treatment or fetal sex (*P* > 0.01) (data not shown). Expression of *AZIN2* and *AGMAT* mRNAs in placentomes was unaffected by P4 treatment (*P* > 0.1) (data not shown).

### Localization of proteins in endometria

**Fig. 3:**
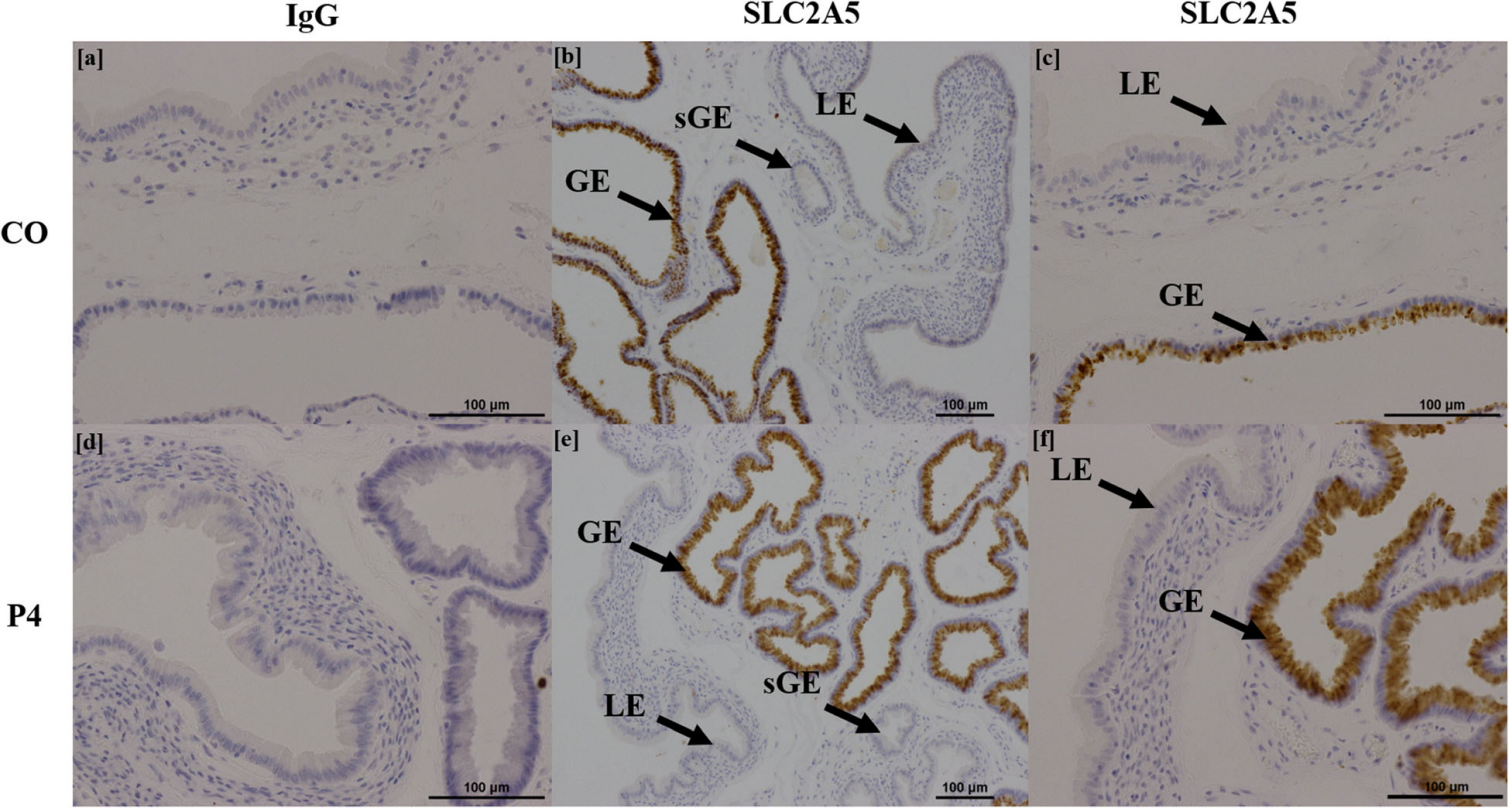
Localization of SLC2A5 protein in endometria of ewes treated with either progesterone (P4) or corn oil (CO). SLC2A5 localized to uterine glandular epithelia (GE) but not uterine luminal (LE) or superficial glandular epithelia (sGE) (**b**, **c**, **e**, **f**). IgG negative controls are provided (**a**, **d**). *n* = 6 animals per treatment group

**Fig. 4:**
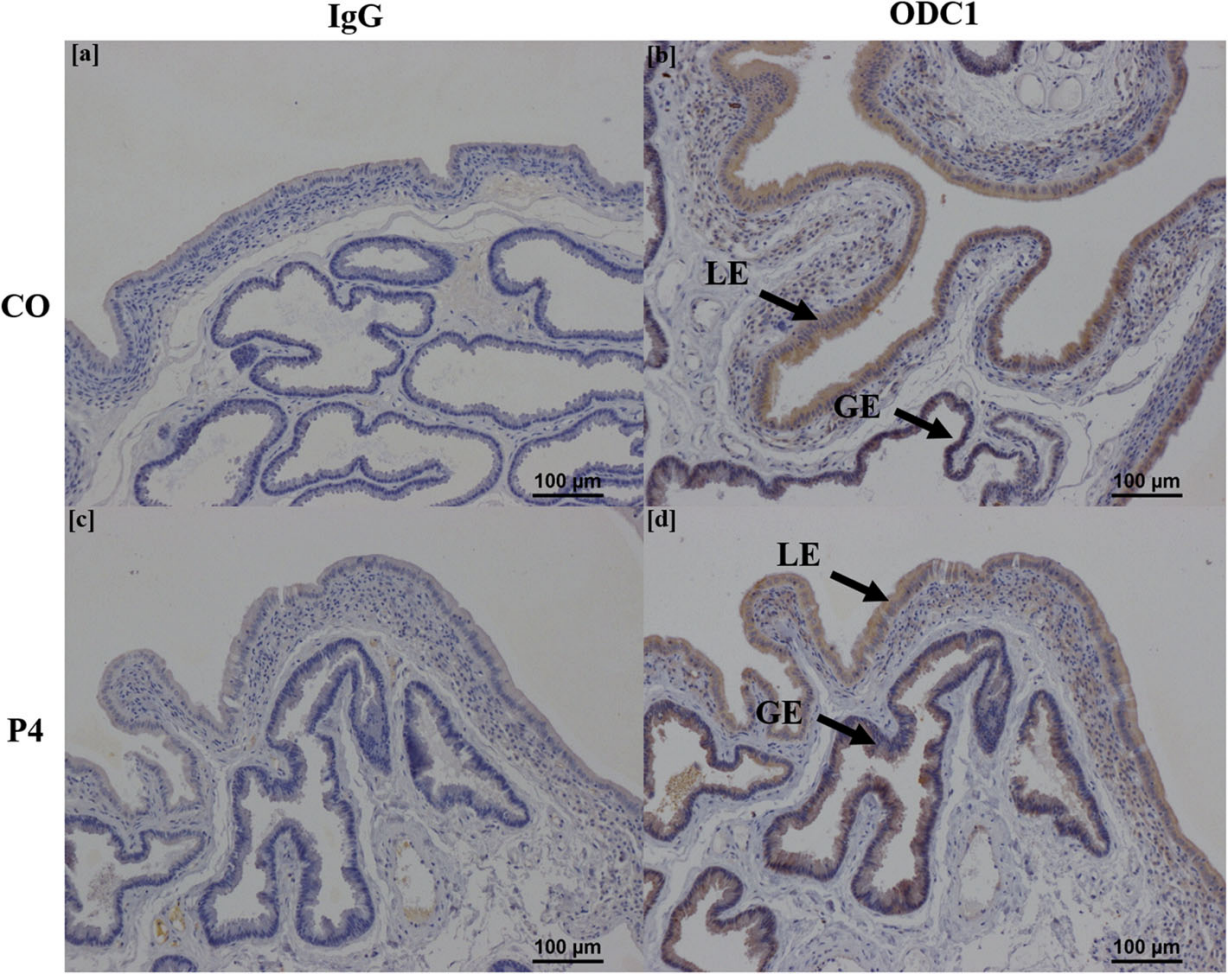
Localization of ODC1 protein in endometria of ewes treated with either progesterone (P4) or corn oil (CO). ODC1 localized to uterine luminal (LE) and glandular epithelia (GE) (**b**, **d**). IgG negative controls are provided (**a**, **c**). *n* = 6 animals per treatment group

**Fig. 5:**
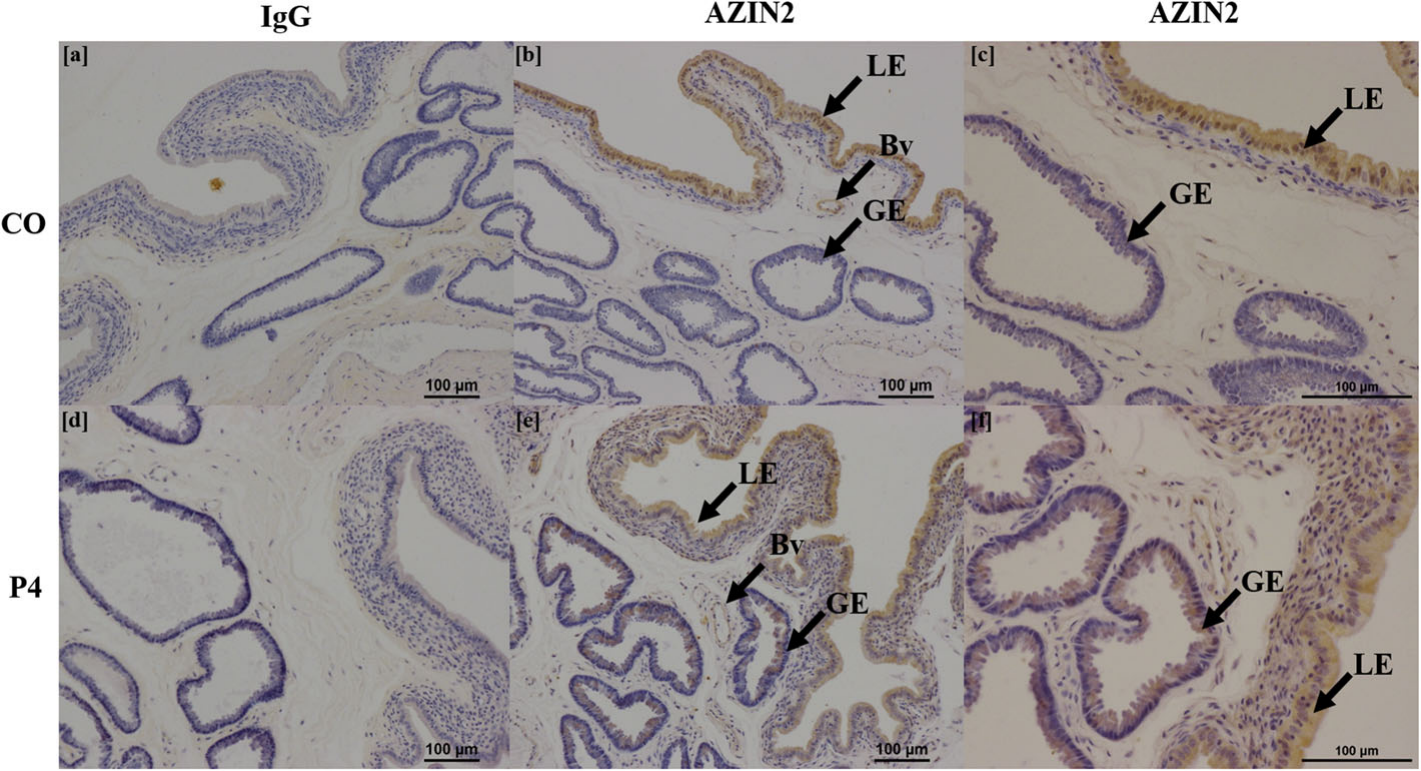
Localization of AZIN2 protein in endometria of ewes treated with either progesterone (P4) or corn oil (CO). AZIN2 localized to uterine luminal epithelia (LE) in CO-treated ewes (**b**), whereas in P4-treated ewes localized to both uterine luminal (LE) and glandular epithelia (GE) (**d**). In both treatment groups, AZIN2 localized to blood vessels (Bv). IgG negative controls are provided (**a**, **c**). *n* = 6 animals per treatment group

**Fig. 6:**
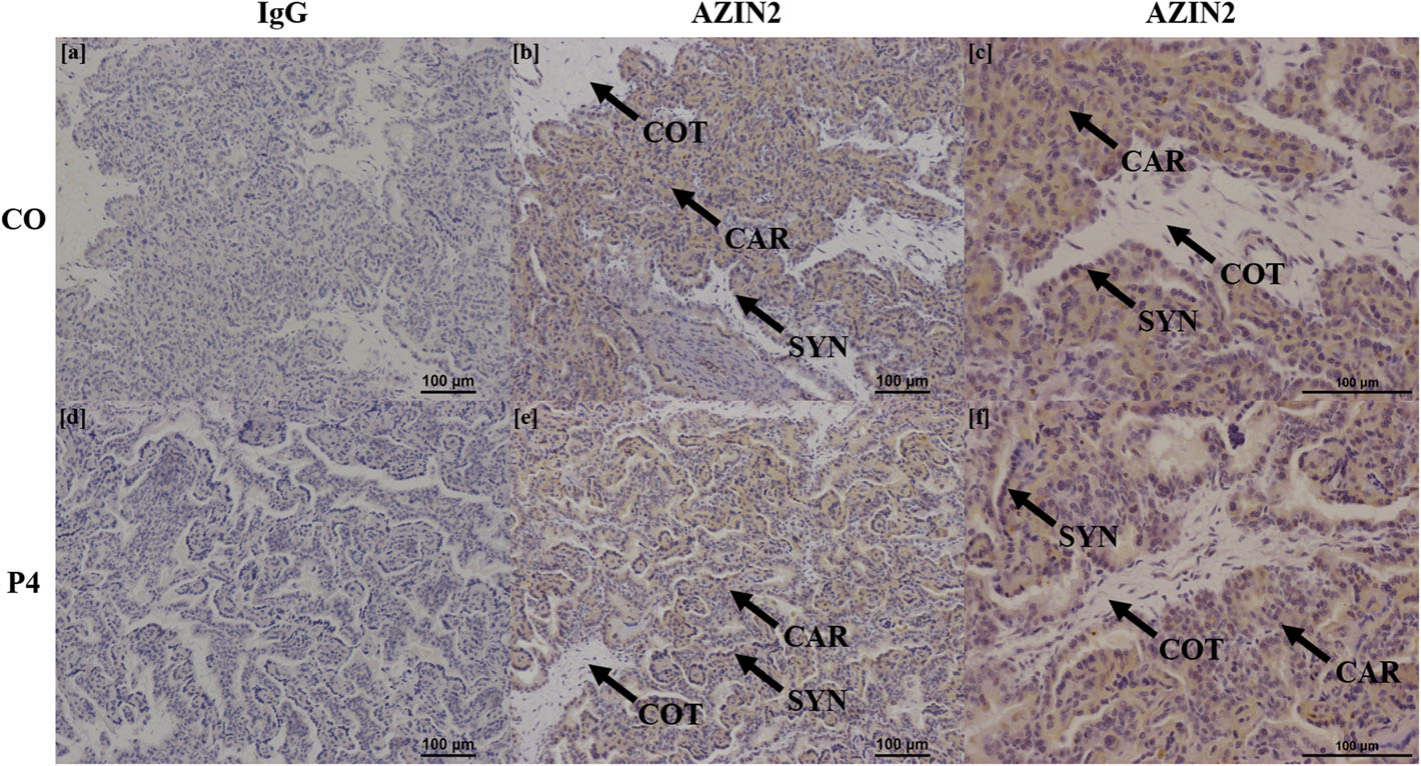
Localization of AZIN2 protein in placentomes of ewes treated with either progesterone (P4) or corn oil (CO). In placentomes, AZIN2 localized to caruncular tissue, syncytialized cells, and mononuclear cells of the chorioallantois. IgG negative controls are provided (**a**, **c**). *n* = 6 animals per treatment group

**Fig. 7:**
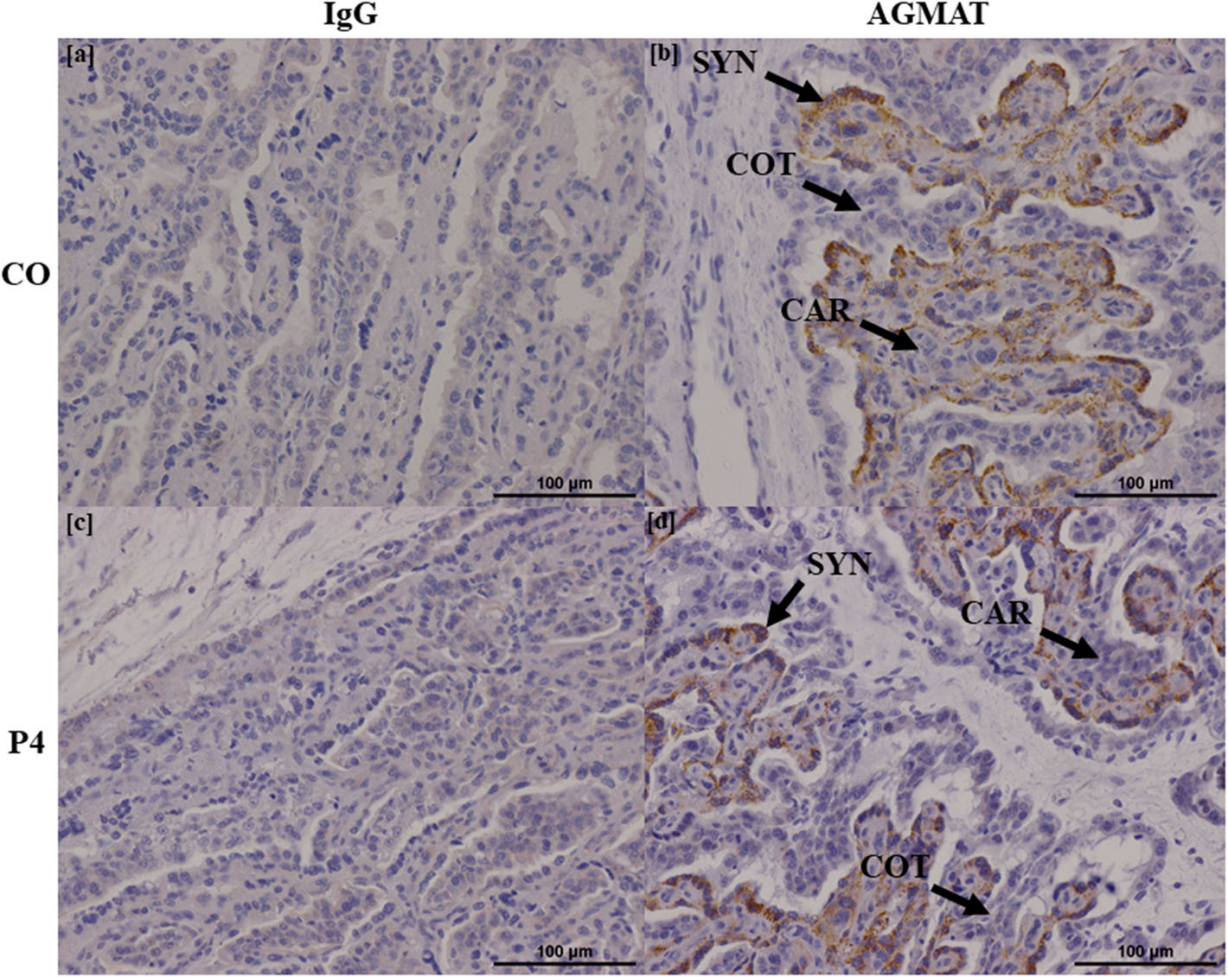
Localization of AGMAT protein in placentomes of ewes treated with either progesterone (P4) or corn oil (CO). AGMAT localized to syncytialized cells within placentomes, but not caruncular or cotyledonary tissue. IgG negative controls are provided (**a**, **c**). *n* = 6 animals per group

Representative images of each protein stained for both CO and P4 treatment groups, as well as IgG controls, are shown in Figs. 3, 4, 5, 6 and 7. SLC2A5 protein localized to uterine glandular epithelia, but not uterine luminal or superficial glandular epithelia (Fig. 3). ODC1 protein localized to both uterine luminal epithelia and glandular epithelia (Fig. 4). AZIN2 positive cells were detected in the uterine luminal epithelia and smooth muscle surrounding blood vessels (Fig. 5). Interestingly, the mean percent of glandular epithelia stained for AZIN2 protein was greater (*P* < 0.05) in P4-treated ewes (22.5 ± 4.8%) compared to CO-treated ewes (4.5 ± 1.8%) (Fig. 5) and localized to uterine luminal epithelia in both treatment groups. AZIN2 protein was widely expressed throughout the placentome including caruncular tissue, syncytialized giant cells, and mononuclear cells of the chorioallanois, but not cotyledonary stroma (Fig. 6). Intriguingly, AGMAT protein localized to syncytialized chorioallanotis in the placentome (Fig. 7).

## Discussion

The results of this study indicate that P4 administration from day 1.5 to day 9 of pregnancy affects abundance of amino acids in placental fluids to 1) increase total aspartate in allantoic fluid; 2) decrease total glutamate in allantoic fluid; and 3) increase total arginine in amniotic fluid. This treatment also influenced the expression of mRNAs in endometrial and placental tissues, suggesting P4-induced modification of these transporters. Previous studies have indicated that progesterone supplementation in early gestation enhances conceptus development [29, 32] however in this study, we determined that treatment of ewes with progesterone in early pregnancy does not increase overall fetal or placental growth at day 125 of gestation.

Interestingly, there was a decrease in pregnancy rates from breeding to determination of pregnancy at day 35 of gestation in the P4-treated group. Other studies demonstrate that asynchronous embryo transfers, particularly if embryos are transferred into a more ‘advanced’ uterine environment, result in reduced pregnancy rates in ruminants [31, 32, 39, 40]. Supplementation of P4 beginning 36 h after the onset of estrus advances the P4-induced up- and down-regulation of genes in the endometrium to create an advanced uterine environment [29] (and Hoskins et al., unpublished results), which likely explains the decreased pregnancy rates observed in this study and in Hoskins et al., unpublished results.

On day 125 of pregnancy, concentrations of P4 in maternal plasma were not different between treatment groups since the P4 supplementation occurred 117 days prior to sample collection. While treatment increased levels of P4 from days 1.5 to 9 of pregnancy (Hoskins et al., unpublished results), P4 concentrations return to endogenous levels by day 12 of pregnancy, 3 days after the last injection (Hoskins et al., unpublished results). Thus, concentrations of P4 reflected only endogenous levels from the placenta [41, 42] and any effects due to treatment at day 125 would have required some ‘programming’ effects of P4 in the first week of gestation, when P4 was administered.

The relationships between P4 and the subsequent uterine responses that, in turn, influence the developing conceptus, have been extensively studied [24, 26, 43]. Early exogenous P4 treatment advances conceptus development in sheep [29, 44], cattle [25, 27], and pigs [45]. These effects of P4-induced accelerated conceptus growth were reported to increase fetal weight and crown rump length when assessed at day 76 of gestation in ewes that received exogenous P4 beginning 36 h after ovulation [31]. Furthermore, fetuses from ewes treated with P4 early in gestation had greater weights of organs such as the brain and heart, in addition to an increase in the surface area of placental membranes [32]. In the present study, there were no detectable differences in fetal or placental parameters of growth at day 125 of pregnancy due to administration of exogenous P4 in early pregnancy. While our findings are not in agreement with results presented by Kleemann and colleagues [31, 32], there are key differences between the two studies. Foremost, in the Kleemann study, tissues were collected on day 76 of pregnancy, which is considered mid-gestation in sheep. At that stage of development, placental growth is sufficient for ovine fetuses to experience an exponential growth phase [46]. Indeed, while fetal growth continues until parturition, the day to day increases are much less in later stages of gestation than they are in mid-gestation [46]. Thus, although phenotypic differences were not observed at day 125 of gestation, this does not mean that there would not have been phenotypic differences earlier in gestation, or even effects on postnatal programming. Further research could involve the described P4 treatment and sampling of tissues at multiple time points across gestation, as well as after parturition.

The caruncular LE and the cotyledonary syncytial epithelial cells within the placentome facilitate the transport of nutrients from the maternal circulation into the fetal-placental circulation and placental fluid compartments via membrane proteins that transport and have affinities for specific nutrients [47, 48]. These cells can regulate nutrient delivery to the fetus by altering the distribution and activities of specific transporters [49]. Nutrients transported from the maternal circulation across the utero-placental interface in the form of histotroph [50] are stored in both allantoic fluid and amniotic fluid, acting as nutrient reserves for the fetus throughout gestation [46]. Our next aim was to determine if early P4 treatment affected maternal and placental tissues on a molecular level to gain insight into possible functional changes in these tissues in response to early P4 treatment. One such measurement of functionality is the composition of nutrients within maternal, fetal, and placental fluids that reflect the ability of the endometrium and placenta to transport nutrients to the fetus. Therefore, we determined the profiles of amino acids, polyamines, and hexose sugars in maternal and fetal plasma, as well as allantoic and amniotic fluids.

Amino acids are unequivocally required by the fetus and placenta for proper growth and development, as amino acids provide functional units for synthesis of DNA, RNA, and proteins [14]. Our results were consistent with those reported by others [3] as glycine was the most abundant amino acid in maternal plasma, while serine, glycine, glutamate, and alanine were the most abundant amino acids in fetal plasma. In the present study, all amino acids were more abundant in fetal plasma than in maternal plasma except for arginine and citrulline. Arginine is not only used as a building block for proteins, but it is also a precursor for important nitrogenous substances including polyamines, agmatine (a substrate for synthesis of polyamines), nitric oxide (NO), and creatine [51, 52]. NO is critical for angiogenesis and vasodilation of the placental vasculature [53], while creatine is important for development of muscle and brain, and generation of ATP [51]. Equivalent concentrations of arginine and citrulline (a precursor of arginine) in maternal and fetal plasma, as compared to all the other amino acids assayed, likely indicates extensive utilization of arginine in support of fetal-placental growth.

Polyamines, downstream metabolites of arginine, are important for normal conceptus development as demonstrated by morpholino knockdown of mRNAs of key enzymes and other proteins involved in the metabolism of arginine to polyamines [35, 54, 55], and the synthesis, secretion, and degradation of polyamines are tightly regulated [9, 56]. While the abundance of polyamines in maternal or fetal plasma was not influenced by P4 treatment, agmatine, the immediate precursor for polyamines, was more abundant than putrescine, spermidine, and spermine in maternal and fetal plasma, as well as in allantoic fluid. Agmatine may serve as a reservoir of substrate for polyamine synthesis as demands of the fetus and placenta increase. Indeed, fetal plasma had a greater abundance of not only agmatine, but also spermine and putrescine compared to maternal plasma, highlighting the higher demand of fetal-placental tissues for polyamines.

While total amounts of aspartate in allantoic fluid tended to be greater in P4-treated ewes, the P4-treated ewes had significantly less glutamate in allantoic fluid than their untreated counterparts. Both aspartate and glutamate are involved in the catabolism of arginine and proline [57] and regulation of the cellular redox state and glycolysis [51, 58]. Interestingly, total amounts of arginine were greater in amniotic fluid of P4-treated ewes, suggesting that P4 may have influenced metabolism and delivery of amino acids from maternal plasma into placental fluid compartments. Indeed, in our study, P4 treatment up-regulated the expression of mRNAs for *SLC7A1* and *SLC7A2* in the endometrium, which implies an increased regulation of the transport of cationic amino acids such as arginine through the SLC7 family of transporters [59]. Further, P4-treated ewes had greater expression of mRNAs for *AGMAT*, a gene encoding agmatinase that is an enzyme that converts agmatine to putrescine that is then metabolized to spermidine and spermine [35], in the endometrium and *ODC1,* a gene encoding ornithine decarboxylase that converts ornithine to putrescine [60], in both the endometrium and placentomes. This suggests increased metabolism of arginine to polyamines for utilization by the fetus and placenta. Moreover, there was a trend for decreased spermidine in amniotic fluid of P4-treated ewes, which may represent increased utilization of spermidine by the fetus. Interestingly, P4 treatment of ewes resulted in an increased expression of AZIN2 protein in the uterine GE compared to CO-treated ewes, suggesting that polyamine synthesis is regulated in some part by P4, and the GE of these animals metabolize arginine via both ODC1- and AZIN2-AGMAT-pathways. Differential localization of the enzymes responsible for synthesis of polyamines highlights the synergy of specific cell types to ensure polyamine delivery to the placenta and fetus.

Expression of mRNAs for *SLC1A4* (neutral amino acid transporter [61]) was up-regulated in placentomes of P4-treated ewes, implying increased regulation of transport of amino acids such as alanine, serine, cysteine, and threonine across the placenta. While the amounts of these amino acids were not different in maternal, fetal, or placental fluids between treatment groups, there is evidence that shows that the placenta itself can utilize serine as a methyl donor for synthesis of purines and thymidine for one-carbon metabolism, and alanine, serine, cysteine, and threonine can be utilized as substrates for gluconeogenesis and other metabolic pathways important for growth and development of the conceptus [14, 62, 63].

Glucose is the primary substrate for synthesis of ATP in most cell types [14] and other metabolic pathways, and is essential for growth and development of the fetal-placental unit in humans [64], rodents [65], and livestock species [66, 67]. P4 administration affects the expression of nutrient transporters, notably those that transport glucose into the uterine lumen [30, 68]. *SLC2A1* (ubiquitous facilitative glucose transporter) mRNA is induced by P4 and further stimulated by IFNT [30, 68], and *SLC2A3* (high affinity facilitative glucose transporter) mRNA is unique to conceptus trophectoderm in early pregnancy and the placenta in mid- to late-pregnancy [68, 69]. In the present study, P4-treated ewes had greater expression of mRNAs for *SLC2A1* in the endometrium. This suggests increased glucose transport from the maternal circulation into the fetal-placental circulation for use, either by the placenta directly, transport into the fetal circulation, or transport across the chorioallantois into the allantoic fluid. Conversely, expression of mRNAs for *SLC2A1* and *SLC2A3* was lower in placentomes of P4-treated ewes. A potential explanation for this alteration could be that placentas from P4-treated ewes actively downregulated these transporters in the placentome due to increased glucose trafficking in the interplacentomal regions of the uterine-placental interface by the *SLC2A1* transporter.

It has been demonstrated that concentrations of glucose in maternal plasma are relatively constant throughout gestation, even as the demands of the fetal-placental unit for glucose increase [46, 70], assuming ewes are well fed and not nutritionally challenged [71, 72]. Given these findings, it is perhaps unsurprising that concentrations of glucose in maternal plasma were not influenced by treatment, the number of fetuses, or fetal sex, as the ewes in this study were not nutrient restricted. Total amounts of glucose in allantoic and amniotic fluid were not different between P4- and CO-treated ewes, despite an up-regulation in *SLC2A1* mRNA in the endometria of P4-treated ewes. This may have been due to increased placental or fetal catabolism of glucose by fetal-placental tissues in P4-treated ewes during pregnancy. Glucose that passes through the fetal-placental circulation may undergo rapid catabolism to produce ATP [73], be converted to fructose which is sequestered within placental tissue and fluids [74, 75], or be metabolized via the pentose phosphate pathway [15, 21, 76], hexosamine biosynthesis pathway [20, 77], or serinogenesis pathway for one-carbon metabolism [22, 78].

Fructose is the most abundant hexose sugar in fetal blood and placental fluids of ungulates and cetaceans [18, 79], and is considered a ‘sequestered’ sugar due to the lack of expression of a transporter to return it back into the maternal circulation [74, 75]. While fructose contributes little to gluconeogenesis and production of ATP [80, 81], it can be used in the hexosamine biosynthesis pathway for the synthesis of glycosaminoglycans such as hyaluronic acid in trophectoderm cells of pigs [21] and sheep [20]. Concentrations of fructose in fetal plasma were significantly greater than those in maternal plasma, which is consistent with results from other studies [18, 79]. In the present study, the expression of mRNAs for *SLC2A5* (transporter of fructose) and *SLC2A8* (transporter of both glucose and fructose) was greater in both endometria and placentomes of P4-treated ewes, but there were no differences in total amounts of fructose in allantoic or amniotic fluid. Potentially, the placentae or fetuses of these P4-treated ewes utilized fructose for hexosamine biosynthesis, which integrates metabolism of amino acids such as glutamine [14], and also stimulates expression of the mechanistic target of rapamycin (mTOR) that activates enzymes for generation of serine for one-carbon metabolism [20, 82]. Indeed, there were differences in concentrations of amino acids in both allantoic and amniotic fluid. Interestingly, the SLC2A5 transporter localized exclusively to uterine glandular epithelia, which indicates that fructose is secreted in histotroph and collects in placental areolae for uptake, rather than directly transported across uterine luminal epithelia to the chorioallantois.

In this study, male fetuses had increased abundances of several amino acids in allantoic fluid and fetal plasma. As most research indicates that male fetuses grow at a faster rate than females [83, 84], the placentas of male fetuses may have been actively transporting and storing greater amounts of amino acids in placental fluids in anticipation for greater demands for fetal growth. More interestingly, placentomes associated with female fetuses had greater expression of the glycine transporter *SLC6A9* than placentomes of male fetuses, suggesting increased regulation of transport of glycine in these placentae. Although glycine was present in greater amounts in allantoic fluid associated with male fetuses, placentae of female fetuses may utilize greater amounts of glycine, rather than storing it in allantoic fluid. It has been suggested that perturbations in placental development in the first trimester of pregnancy affect females more than males [84], and that placental tissues express genes in a sexually dimorphic manner [85]. In humans, a pathway analysis revealed sex-biased genes involved in mTOR signaling in placentae [86], a biologically relevant pathway within the placenta [77, 87]. mTOR signaling activates enzymes for generation of serine for one-carbon metabolism [22, 51], and also integrates metabolism of amino acids and hexose sugars (particularly fructose) to regulate nutrient and growth signaling within the fetal-placental unit [20, 77, 87]. In accordance with this, greater expression of the fructose transporters *SLC2A5* and *SLC2A8* were observed in placentomes of female fetuses, suggesting that placentae of female fetuses had a greater capacity to transport fructose. Further studies are required to determine sex-specific pathways of amino acid utilization within the fetal-placental unit.

## Conclusions

Collectively, results of the present study identified P4-induced modifications in the endometrium and placenta that were influenced by treatment of ewes with P4 in early gestation, even before signaling for maternal recognition of pregnancy (as discussed by the companion paper to this study, Hoskins et al. 2020). These modifications influenced the expression of mRNAs for nutrient transporters in fetal-placental tissues, the expression of a key protein in uterine glandular epithelia, and composition of placental fluids, thereby influencing the pool of nutrients available to support development of the fetal-placental unit as pregnancy progressed. Further studies are necessary to determine how treatment with P4 in early gestation affects developing fetal tissues, and how those effects may influence neonatal performance. Characterizing how exogenous steroids affect the gestational environment ultimately aids in our understanding of how hormonal regimens, used either in animal production or in human medicine, influence development of the conceptus and successful outcomes of pregnancy.

## Supplementary Information

**Additional file 1: Supplementary Fig. 1** Relative abundances of amino acids in maternal and fetal plasma (a,b) and placental fluids (c,d). Amino acids in plasma are expressed as concentrations (nmol/mL), and amino acids in placental fluids are expressed as total amounts (concentration × volume). Glutamate, glycine, and alanine were most abundant in maternal plasma (a), while glutamate, serine, glycine, and alanine were most abundant in fetal plasma (b). All amino acids, except for arginine and citrulline, were more abundant in fetal plasma than maternal plasma. The most abundant amino acids in allantoic fluid were serine and taurine (c). The most abundant amino acids in amniotic fluid were asparagine and serine (d).

**Additional file 2: Supplementary Fig. 2** Relative abundances of polyamines in maternal and fetal plasma (a,b) and placental fluids. Agmatine and polyamines in plasma are expressed as concentrations (nmol/mL), and expressed as total amounts (concentration × volume) in placental fluids. Agmatine was most abundant in the plasma of both ewes and fetuses (a,b). Similarly, agmatine was most abundant in allantoic fluid (c). In amniotic fluid, total amounts of agmatine were similar to those for polyamines, except for putrescine, which was least abundant (d).

**Additional file 3: Supplementary Fig. 3** Relative abundances of hexose sugars in maternal and fetal plasma (a,b) and placental fluids (c,d). Glucose and fructose concentrations in plasma are expressed as concentrations (μmol/L), and in placental fluids are expressed as total amounts (concentration × volume). There were greater concentrations of glucose than fructose in maternal plasma (a), in contrast to fetal plasma (b) which had greater concentrations of fructose compared to glucose. Similarly, both allantoic fluid (c) and amniotic fluid (d) had greater total amounts of fructose compared to glucose. Mean values and SEM are presented. *n* = 5–9 samples per group.

**Additional file 4: Supplementary Table 1.** Primers used for real-time qPCR

**Additional file 5: Supplementary Table 2.** Antibodies used for immunohistochemistry

**Additional file 6: Supplementary Table 3**. A summary of measurements affected by progesterone (P4) treatment, pregnancy type, and fetal sex. Includes interactions that are significant (*P* < 0.05) or tend to be significant (*P* < 0.1).

## Data Availability

The datasets generated in the current study can be made available from the corresponding author upon reasonable request.

## Abbreviations

ANOVA: Analysis of variance
ATP: Adenosine triphosphate
CO: Corn oil
CT: Cycle threshold
GE: Uterine glandular epithelia
HPLC: High performance liquid chromatography
IFNT: Interferon-tau
LE: Uterine luminal epithelial epithelia
MTOR: Mechanistic target of rapamycin
NADPH: Nicotinamide adenine dinucleotide phosphate hydrogen
NO: Nitric oxide
OPA: *o*-Phthaldialdehyde
P4: Progesterone
PBS: Phosphate buffered saline
RIN: RNA integrity number
SEM: Standard error of the mean
sGE: Uterine superficial glandular epithelia
qPCR: Quantitative polymerase chain reaction
