# Supplementary figures and images for: Pre-implantation exogenous progesterone and pregnancy in sheep. II. Effects on fetal-placental development and nutrient transporters in late pregnancy
Source: J Anim Sci Biotechnol. 2021 Apr 8;12:46. doi: 10.1186/s40104-021-00567-1 (PMC8028684; doi:10.1186/s40104-021-00567-1)

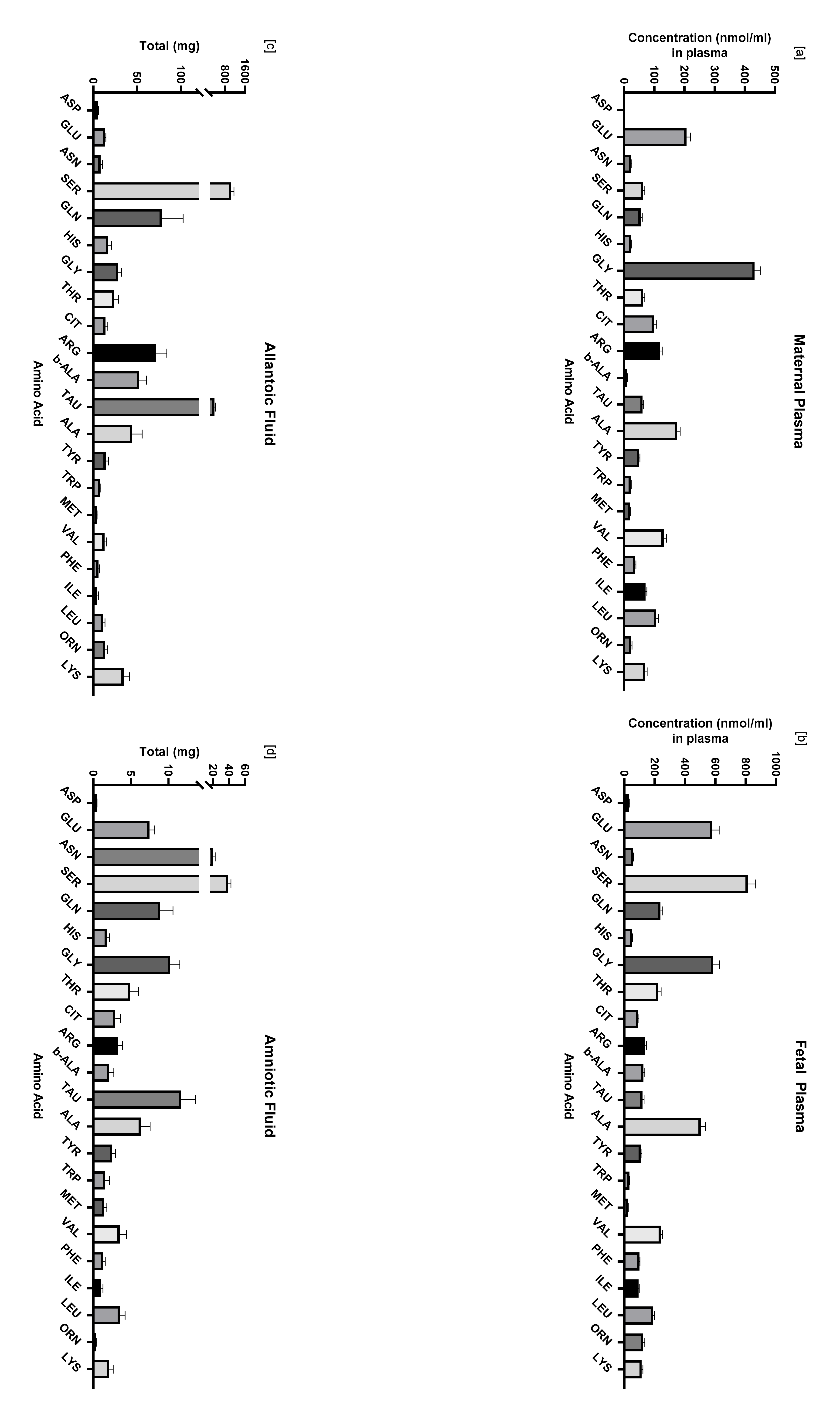

Supplement: Supplementary file 1 — Additional file 1: Supplementary Fig. 1 Relative abundances of amino acids in maternal and fetal plasma (a,b) and placental fluids (c,d). Amino acids in plasma are expressed as concentrations (nmol/mL), and amino acids in placental fluids are expressed as total amounts (concentration × volume). Glutamate, glycine, and alanine were most abundant in maternal plasma (a), while glutamate, serine, glycine, and alanine were most abundant in fetal plasma (b). All amino acids, except for arginine and citrulline, were more abundant in fetal plasma than maternal plasma. The most abundant amino acids in allantoic fluid were serine and taurine (c). The most abundant amino acids in amniotic fluid were asparagine and serine (d). [file 40104_2021_567_MOESM1_ESM.tif]

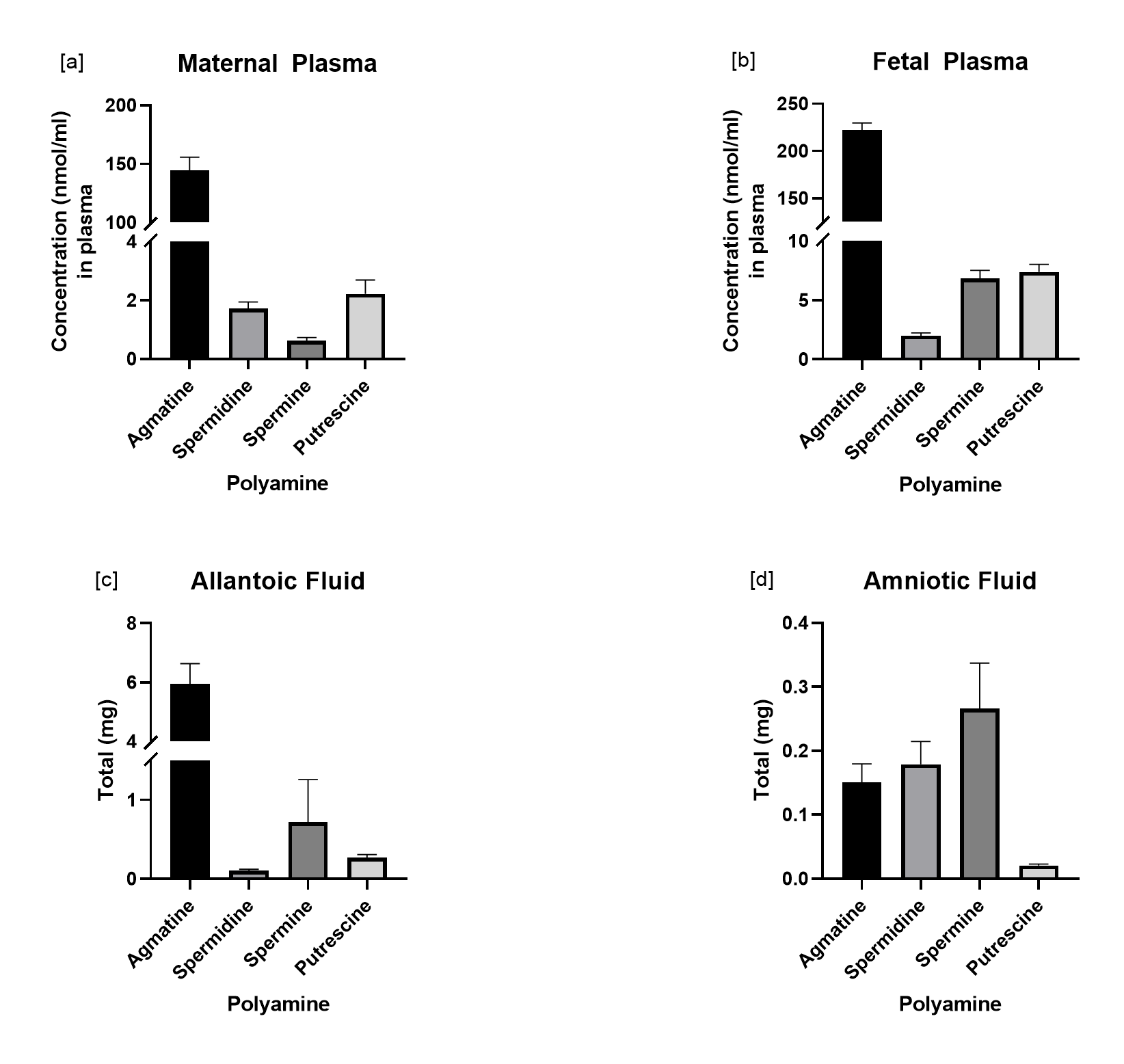

Supplement: Supplementary file 2 — Additional file 2: Supplementary Fig. 2 Relative abundances of polyamines in maternal and fetal plasma (a,b) and placental fluids. Agmatine and polyamines in plasma are expressed as concentrations (nmol/mL), and expressed as total amounts (concentration × volume) in placental fluids. Agmatine was most abundant in the plasma of both ewes and fetuses (a,b). Similarly, agmatine was most abundant in allantoic fluid (c). In amniotic fluid, total amounts of agmatine were similar to those for polyamines, except for putrescine, which was least abundant (d). [file 40104_2021_567_MOESM2_ESM.tif]

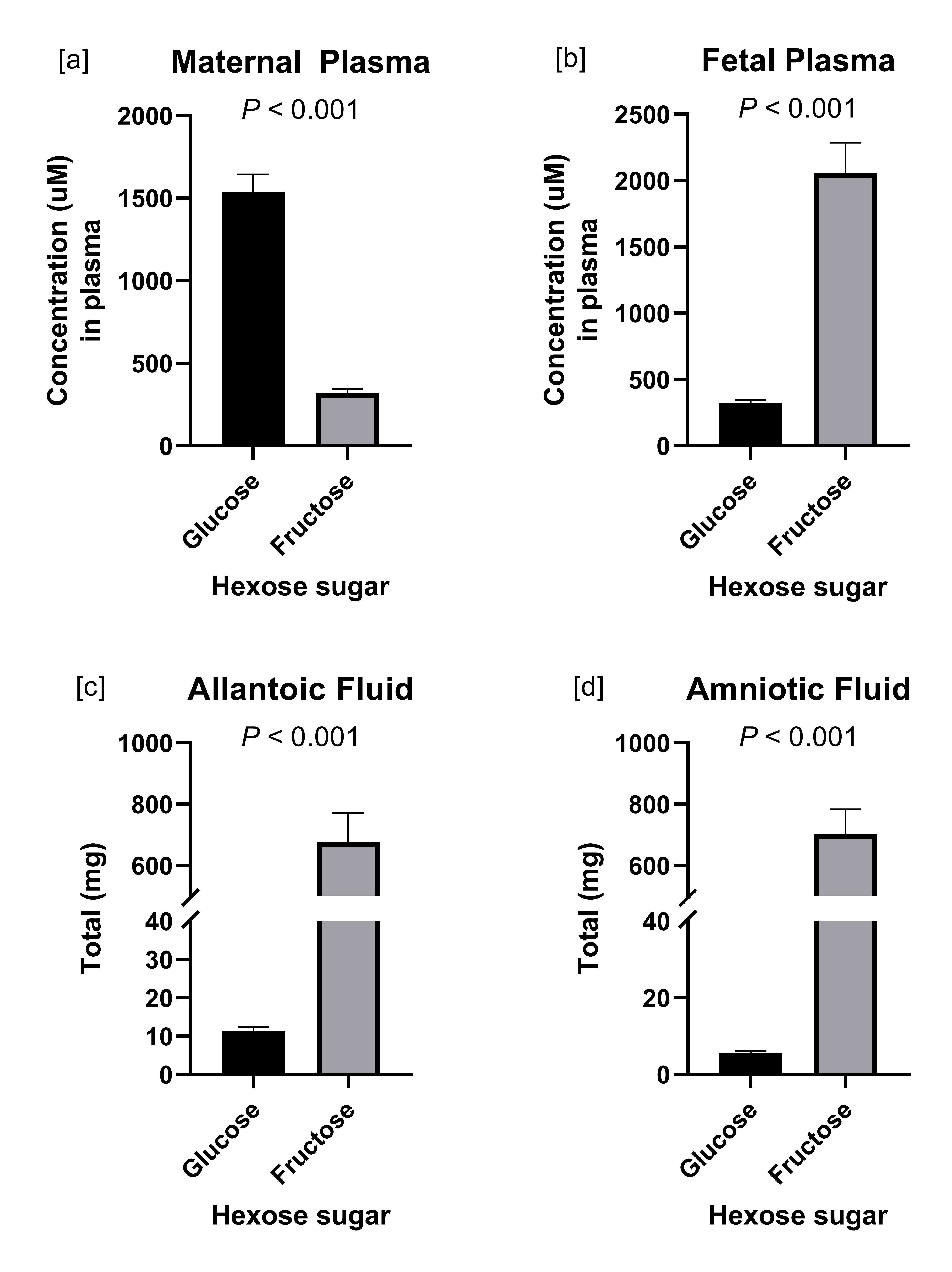

Supplement: Supplementary file 3 — Additional file 3: Supplementary Fig. 3 Relative abundances of hexose sugars in maternal and fetal plasma (a,b) and placental fluids (c,d). Glucose and fructose concentrations in plasma are expressed as concentrations (μmol/L), and in placental fluids are expressed as total amounts (concentration × volume). There were greater concentrations of glucose than fructose in maternal plasma (a), in contrast to fetal plasma (b) which had greater concentrations of fructose compared to glucose. Similarly, both allantoic fluid (c) and amniotic fluid (d) had greater total amounts of fructose compared to glucose. Mean values and SEM are presented. n = 5–9 samples per group. [file 40104_2021_567_MOESM3_ESM.tif]
