# Supplementary material for: Pre-implantation exogenous progesterone and pregnancy in sheep. II. Effects on fetal-placental development and nutrient transporters in late pregnancy
Source: J Anim Sci Biotechnol. 2021 Apr 8;12:46. doi: 10.1186/s40104-021-00567-1 (PMC8028684; doi:10.1186/s40104-021-00567-1)
Supplement: Supplementary file 4 — Additional file 4: Supplementary Table 1. Primers used for real-time qPCR [file 40104_2021_567_MOESM4_ESM.docx]

| **Supplementary Table 1.** Primers used for real-time qPCR | |  |  |  |
| --- | --- | --- | --- | --- |
| Gene abbrev. | Gene name | Forward/reverse primers (5'→3') | Product size, bp | GenBank accession no. |
| *SLC7A1* | Solute carrier family 7, member 1 | F: CCTAGCGCTCCTGGTCATCA | 85 | AF212146 |
|  |  | R: GGGCGTCCTTGCCAAGTA |  |  |
| *SLC7A2* | Solute carrier family 7, member 2 | F: GCAGAGCAGCGCTGTCTTT | 88 | XM_010820288.3 |
|  |  | R: ACTGTCCAGAGTGACGATTTTCC |  |  |
| *SLC6A9* | Solute carrier family 6, member 9 | F: CGCTTCGTCTCACCAGCTAT | 126 | NM_001242343.1 |
|  |  | R: CAGAGCCATGAGGAAGCCAA |  |  |
| *SLC1A4* | Solute carrier family 1, member 4 | F: GTTGCTATCACCCAAGCCCT | 101 | NM_001081577.1 |
|  |  | R: AGGGGAGCTTCACTGTCTCT |  |  |
| *SLC2A1* | Solute carrier family 2, member 1 | F: TGGGAAAGTCCTTTGAGATGC | 107 | NM_174602.2 |
|  |  | R: GGTCAGGCCGCAGTACACA |  |  |
| *SLC2A3* | Solute carrier family 2, member 3 | F: AAATTAGGGCCATGGGGACCA | 98 | NM_174603.3 |
|  |  | R: TTTTATGATCGCCTCAGGAGCA |  |  |
| *SLC2A5* | Solute carrier family 2, member 5 | F: GGTGGGAATATGTGCAGGTC | 154 | NM_001009451.1 |
|  |  | R: CAGTCAATCCGAGGAGGATGG |  |  |
| *SLC2A8* | Solute carrier family 2, member 8 | F: CGTCCTCACCAACTGGTTCA | 197 | NM_201528.1 |
|  |  | R: CCCTTTGGTCTCAGGGACAC |  |  |
| *ODC1* | Ornithine decarboxylase | F: GCACATCCAAAGGCCAAGTT | 92 | M92441.1 |
|  |  | R: GGCGACAGACTGCTTTGGAA |  |  |
| *AZIN2* | Antizyme inhibitor 2 (also known as *ADC* [arginine decarboxylase]) | F: GCTGTGGTGTGGACATCCTT | 161 | NM_001293722.1 |
|  |  | R: ACTGAGGTGGTACACGATGC |  |  |
| *AGMAT* | Agmatinase | F: GATGGCTTGGACCCTGCTTA | 98 | XM_004013796.4 |
|  |  | R: CCTTGACAACCCCGGATGAT |  |  |
| *SDHA* | Succinate dehydrogenase complex, subunit A | F: CATCCACTACATGACGGAGCA | 77 | AY970969.1 |
|  |  | R: ATCTTGCCATCTTCAGTTCTGCTA |  |  |
| *GAPDH* | Glyceraldehyde-3-phosphate dehydrogenase | F: GGGCAGCCCAGAACATCAT | 112 | NM_001190390.1 |
|  |  | R: CCAGTGAGCTTCCCGTTCAG |  |  |
