# Supplementary material for: Pre-implantation exogenous progesterone and pregnancy in sheep. II. Effects on fetal-placental development and nutrient transporters in late pregnancy
Source: J Anim Sci Biotechnol. 2021 Apr 8;12:46. doi: 10.1186/s40104-021-00567-1 (PMC8028684; doi:10.1186/s40104-021-00567-1)
Supplement: Supplementary file 5 — Additional file 5: Supplementary Table 2. Antibodies used for immunohistochemistry [file 40104_2021_567_MOESM5_ESM.docx]

| **Supplementary Table 2.** Antibodies used for immunohistochemistry | | | |  |  |  |  |
| --- | --- | --- | --- | --- | --- | --- | --- |
| Protein Abbrev. | Protein name | Function | Manufacturer | Product number | Antibody  information | Dilution | Antigen retrieval |
| SLC2A5 | Solute carrier family 2, member 5 | Transporter for fructose only | Sigma-Aldrich | AV42096 | Rabbit;  Polyclonal | 1:100 | 0.1 mol/L boiling citrate buffer (10 min) |
| ODC1 | Ornithine decarboxylase | Removes carboxylic acid group from ornithine to form putrescine | Abcam | ab97395 | Rabbit;  Polyclonal | 1:450 | 0.1 mol/L boiling citrate buffer (10 min) |
| AZIN2 | Antizyme inhibitor 2 (also known as ADC [arginine decarboxylase]) | Removes carboxylic acid group from arginine to form agmatine | Abcam | ab192771 | Rabbit;  Polyclonal | 1:350 (endometria) 1:250 (placentomes) | 0.5 mg/mL protease in 1× PBS (8 min) |
| AGMAT | Agmatinase | Catalyzes reaction with agmatine and water to form putrescine and urea | Abcam | ab231894 | Rabbit;  Polyclonal | 1:100 | 0.1 mol/L boiling citrate buffer (10 min) |
