# Supplementary material for: Pre-implantation exogenous progesterone and pregnancy in sheep. II. Effects on fetal-placental development and nutrient transporters in late pregnancy
Source: J Anim Sci Biotechnol. 2021 Apr 8;12:46. doi: 10.1186/s40104-021-00567-1 (PMC8028684; doi:10.1186/s40104-021-00567-1)
Supplement: Supplementary file 6 — Additional file 6: Supplementary Table 3. A summary of measurements affected by progesterone (P4) treatment, pregnancy type, and fetal sex. Includes interactions that are significant (P < 0.05) or tend to be significant (P < 0.1). [file 40104_2021_567_MOESM6_ESM.docx]

**Supplementary Table 3**. A summary of measurements affected by progesterone (P4) treatment, pregnancy type, and fetal sex. Includes interactions that are significant (*P*<0.05) or tend to be significant (*P*＜0.1).

| Parameter | Effect | | |
| --- | --- | --- | --- |
|  | Treatment  (CO vs. P4) | Pregnancy type  (Singleton vs. Twin) | Fetal sex  (Male vs. Female) |
| Fetal and placental phenotypes |  | - Placenta length^c^  - Placentome number^c^ |  |
| Maternal plasma |  | - Amino acids   - Aspartate^c^ - Threonine^c^ - Arginine^c^ - Valine^c^ - Ornithine^c^ - Citrulline^c^ - Glutamate^d^   - Agmatine and polyamines   - Spermidine^c^ |  |
| Fetal plasma | - Agmatine and polyamines   - Putrescine^a+^ | - Amino acids   - Threonine^c^ - B-alanine^c^ - Tryptophan^c^ - Methionine^c^ - Valine^c^ - Aspartate^d^ - Serine^d^ - Glycine^d^   - Agmatine and polyamines   - Putrescine^c+^ - Spermine^c^ | - Amino acids   - Threonine^e^   - Agmatine and polyamines   - Spermine^e^ |
| Allantoic fluid | - Amino acids   - Glutamate^a^ - Aspartate^b^ | - Amino acids   - Taurine^c^   - Agmatine and polyamines   - Spermidine^d^ | - Amino acids   - Serine^e^ - Glutamine^e-^ - Histidine^e-^ - Glycine^e-^ - Threonine^e-^ - Arginine^e-^ - Taurine^e-^ - Tryptophan^e-^ - Phenylalanine^e^ - Isoleucine^e^ - Leucine^e-^   - Agmatine and polyamines   - Agmatine^e^ |
| Amniotic fluid | -Amino acids   - Arginine^b^ | - Amino acids   - Lysine^c^ - Tryptophan^c^   - Agmatine and polyamines   - Agmatine^d^   -Hexose sugars   - Glucose^c^ | - Amino acids   - Ornithine^f^   - Agmatine and polyamines   - Spermidine^e^ |
| mRNAs | - Endometria   - *SLC7A1*^b^ - *SLC7A2*^b^ - *SLC2A1*^b^ - *SLC2A5*^b^ - *ODC1*^b^ - *AGMAT*^b^   - Placentomes   - *SLC1A4*^b^ - *SLC2A5*^b^ - *SLC2A8*^b^ - *SLC2A1*^a^ - *SLC2A3*^a^ | - Endometria   - *AZIN2*^d^   - Placentomes   - *SLC7A1*^c^ - *SLC7A2*^c^ - *SLC2A1*^c^ - *SLC2A3*^c^ | - Placentomes   - *SLC6A9*^f^ - *SLC2A5*^f^ - *SLC2A8*^f^ |
| Proteins | - Endometria   - AZIN2^b^ |  |  |

Legend:

^a^: CO ˃ P4

^b^: P4 ˃ CO

^c^: Singleton ˃ Twin

^d^: Twin ˃ Singleton

^e^: Male ˃ Female

^f^: Female ˃ Male

^+^: interaction of treatment × pregnancy type

^－^: interaction of treatment × fetal sex
